# Dominant intragraft plasma cells targeting bilirubin implicate local heme catabolism in human cardiac allograft vasculopathy

**DOI:** 10.1172/JCI194138

**Published:** 2025-11-25

**Authors:** Sarah B. See, Talita Aguiar, Max Dietzel, Mattea Ausmeier, Hang T.T. Nguyen, Shunya Mashiko, Laura Donadeu, Hector Cordero, Poulomi Roy, Lorea Roson, Charles C. Marboe, Matthias J. Szabolcs, Maryjane Farr, Jose González-Costello, Aleix Olivella, Yoshifumi Naka, Koji Takeda, Rodica Vasilescu, Kevin J. Clerkin, Gilles Benichou, Joren C. Madsen, R. Glenn King, Oriol Bestard, Evan P. Kransdorf, Emmanuel Zorn

**Affiliations:** 1 Columbia University Irving Medical Center, New York, New York, USA.; 2 Institute of Anatomy and Cell Biology, Faculty of Medicine, Martin-Luther-University Halle-Wittenberg, Halle, Germany.; 3 Kidney Transplant Unit, Nephrology Department, Vall d’Hebron University Hospital, Barcelona, Spain.; 4 Immunology Unit, Department of Physiology, University of Extremadura, Caceres, Spain.; 5 University of Texas Southwestern Medical Center, Dallas, Texas, USA.; 6 Department of Cardiology, Hospital Universitari de Bellvitge, BIOHEART cardiovascular diseases research group, IDIBELL, University of Barcelona, CiberCV, Barcelona, Spain.; 7 Heart Failure Unit, Department of Cardiology, Hospital Universitari Vall d’Hebrón, Vall d’Hebrón Institut de Recerca (VHIR), Universitat Autònoma de Barcelona, CIBERCV, Instituto de Salud Carlos III, Madrid, Spain.; 8 Massachusetts General Hospital, Boston, Massachusetts, USA.; 9 University of Alabama, Birmingham, Alabama, USA.; 10 Smidt Heart Institute Cedars-Sinai Medical Center, Los Angeles, California, USA.

**Keywords:** Cardiology, Immunology, Transplantation

## Abstract

**BACKGROUND:**

Cardiac allograft vasculopathy (CAV) is consistently accompanied by immune infiltrates surrounding affected coronary arteries, including antibody-producing plasma cells (PC). The antigenic drivers of these intragraft PC responses remain poorly defined.

**METHODS:**

We characterized graft infiltrating PC by single-cell RNA sequencing and immunoglobulin gene profiling. Using immunoglobulin sequences, we generated 37 recombinant monoclonal antibodies (mAb) from dominant intragraft PC clones and 24 control mAb from peripheral blood PC. Antigen reactivity was screened against chemical adducts, including bilirubin, a heme-degradation byproduct. Histologic and tissue analyses assessed bilirubin deposition as well as expression of hemecatabolic enzymes and the presence of Fe^2+^ in heart explants with CAV.

**RESULTS:**

A majority of graft-derived mAb (21 of 37; approximately 57%) — but none of the mAb derived from blood PC — reacted to bilirubin. Bilirubin deposition was detected within lymphocytic aggregates in CAV grafts. In coronary arteries with CAV lesions, bilirubin accumulated in the cytoplasm and nuclei of smooth muscle cells in the tunica media, a pattern not observed in healthy heart tissue. Lastly, we detected the expression of heme-oxygenase-1 and biliverdin reductases in graft-infiltrating macrophages along with the presence of Fe^2+^ ion in the media of arteries with hyperplasia.

**CONCLUSION:**

These findings suggest that local heme catabolism and resultant bilirubin accumulation create a prominent target for intragraft antibody responses in CAV. Bilirubin-specific antibodies and hemecatabolic pathways may contribute to CAV pathogenesis and represent potential mechanistic and therapeutic avenues for further investigation.

**FUNDING:**

Grants AI116814, AI154845, AI184963, and AI176507, HL148528, RYC2022-036797-I, P30CA013696, UL1TR001873, S10OD020056.

## Abstract 2

Plasma cells in human cardiac allografts with vasculopathy target bilirubin, revealing local heme catabolism and suggesting a novel contributor to CAV pathogenesis.

## Graphical abstract

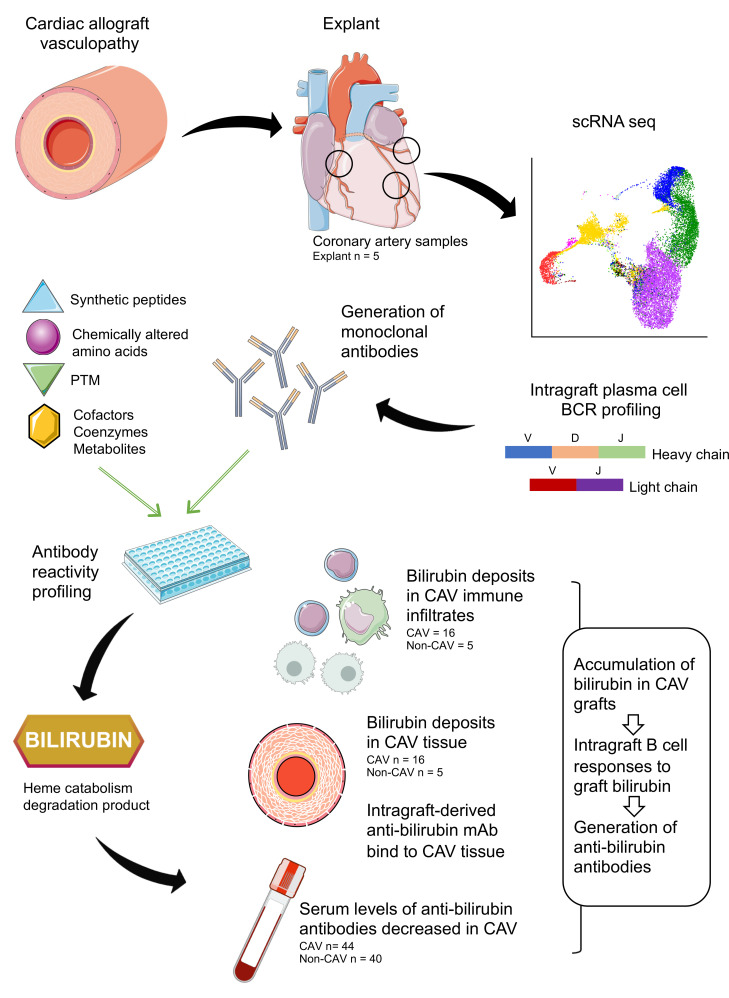

## Introduction

Cardiac allograft vasculopathy (CAV) is a leading cause of mortality following heart transplantation. Treatment options for CAV are limited, and retransplantation is often the sole recourse, but is only available in a minority of patients who meet stringent criteria to be added back to the waitlist. A considerable challenge in developing new treatments is our incomplete understanding of CAV’s underlying mechanisms, which has resulted in only incremental advances, despite decades of research. Clinical observations have documented the presence of dense B cell infiltrates around coronary arteries in transplanted hearts with CAV, a finding confirmed by 3 independent research groups (1–4). These infiltrates contain both memory B cells and plasma cells (PCs) that occasionally organize into structures resembling germinal centers, called tertiary lymphoid organs (TLOs). Importantly, such infiltrates are absent in control native hearts or in transplanted hearts without CAV, suggesting that PCs may play a key role in CAV pathophysiology.

**Figure 1:**
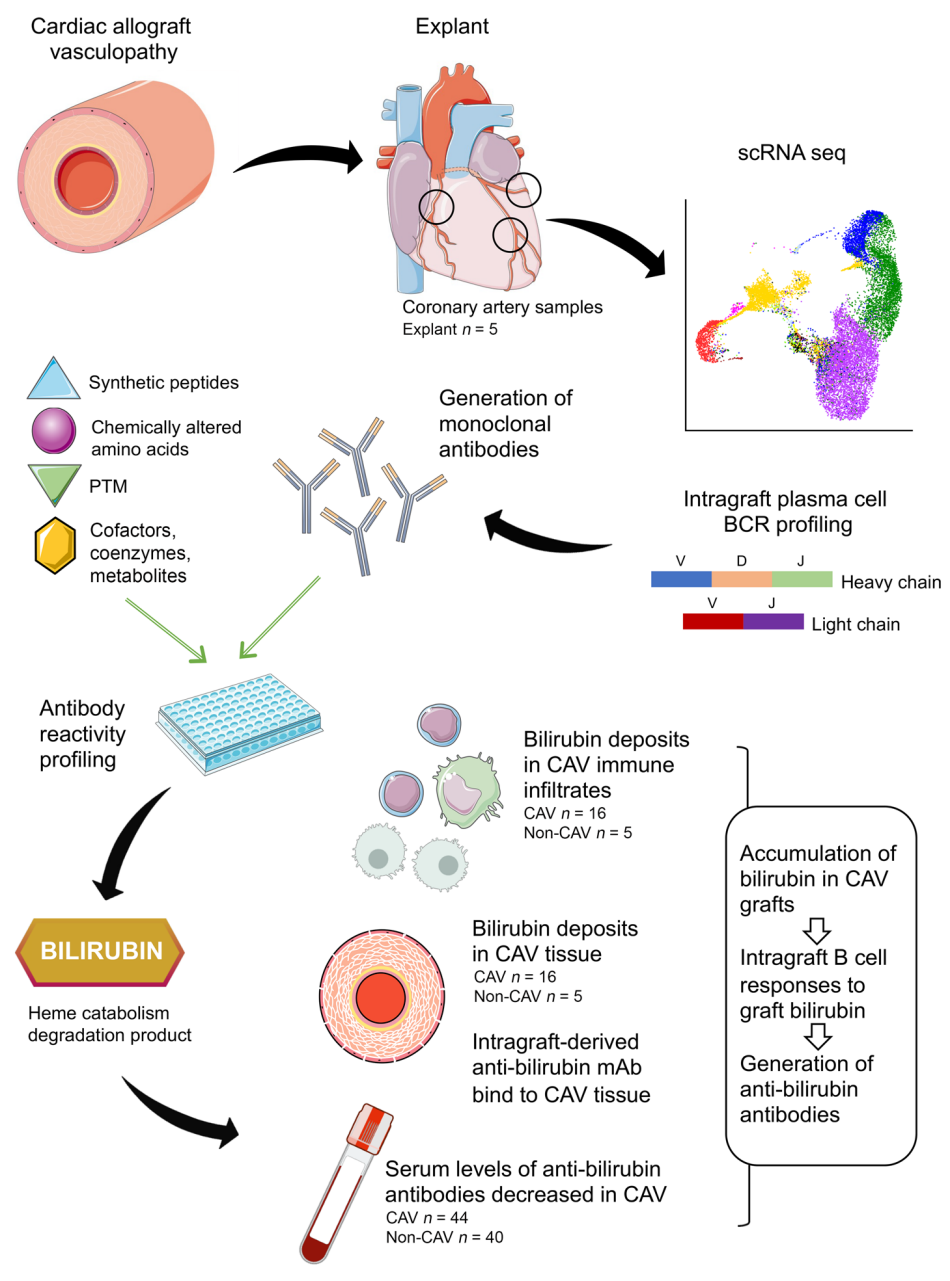
Flow diagram of the study design.

In previous studies, we used graft specimens with CAV, which were explanted at the time of retransplantation to investigate infiltrating B cells. Analysis of the in situ rearranged immunoglobulin heavy chain variable region (IGHV) repertoire revealed robust expansion and somatic mutation of B cell clones at different locations in 4 out of 4 cardiac allografts studied, with no comparable expansion in paired peripheral blood (5). We subsequently derived over 100 immortalized B cell clones from the graft tissue to assess the specificity of infiltrating CD20^+^ B cells (1). None of these infiltrating B cells was specific to donor human leukocyte antigen (HLA), which was consistent with the lack of association between B cell, PC infiltrates, and circulating donor-specific antibodies (DSA) or antibody-mediated rejection (ABMR) in CAV (1, 4). However, we found a predominance of innate-like B cells with a polyreactive profile (1). We recently reported that polyreactivity can often be explained by the recognition of shared chemical moieties exposed on different molecules such as posttranslational modifications or other adducts (6). This observation led us to hypothesize that graft-infiltrating B cells and PCs with polyreactive profiles may also recognize chemical adducts in CAV. In the present study (outlined in Figure 1), we test this hypothesis by examining intragraft antibody responses associated with CAV focusing primarily on dominant PC clones.

## Results

### Composition of immune infiltrates in CAV.

**Figure 2:**
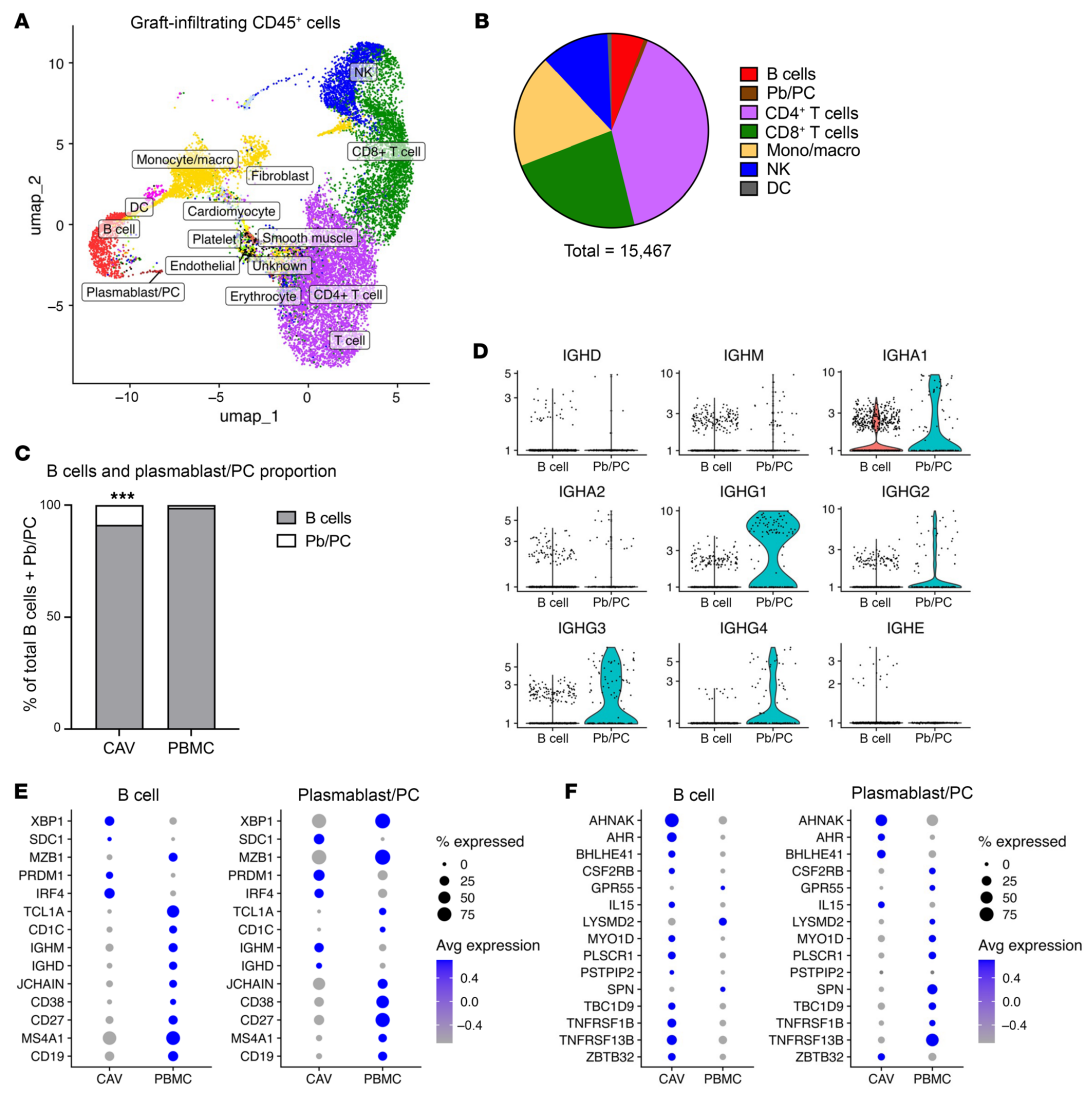
Innate-like IgG-switched plasmablasts/PCs are enriched in graft-infiltrates in CAV. (**A**) Uniform manifold approximation and projection (UMAP) embedding of integrated scRNAseq data obtained for graft-infiltrating CD45^+^ cell populations in 5 explants with CAV. (**B**) Composition of intragraft immune cell populations in 5 explants with CAV. (**C**) Proportion of intragraft B cells and plasmablasts/plasma cells in CAV and healthy donor PBMC. (**D**) Distribution of immunoglobulin heavy chain isotypes of B cells and plasmablast/plasma cells in CAV. (**E**) Expression signatures of B cell and plasmablast-specific markers in CAV and healthy donor PBMC. (**F**) Expression signatures of specific innate-like B cell markers in CAV and healthy donor PBMC. Statistical analysis was performed by Fisher’s exact test and significance depicted as ***P < 0.001.

To characterize the composition of immune cells associated with CAV, we isolated CD45^+^ cells from dissociated cardiac tissue surrounding the coronary arteries from 5 explanted allografts obtained at time of retransplantation. All 5 patients experienced heart failure due to CAV, justifying retransplant. Single-cell RNA-seq (scRNAseq) cluster analysis and pseudotemporal trajectory analysis of infiltrating cells identified 5 main subsets present in all 5 explants, as illustrated in Figure 2A and Supplemental Figure 1 (supplemental material available online with this article; https://doi.org/10.1172/JCI194138DS1). CD4^+^ (40%) and CD8^+^ (23%) T cells were the most abundant cells followed by monocytes/macrophages (18.9%), NK (11.3%) cells, B cells (5.4%), and PCs (0.7%) (Figure 2B). A comparison of graft infiltrate composition with peripheral blood B cell subsets revealed a significant enrichment of PCs within the graft (8.82%) compared with blood PCs (1.22%), supporting the hypothesis of localized antibody responses in CAV (Figure 2C). Immunoglobulin isotype analysis demonstrated the predominance of IgG1-producing cells within intragraft PCs, with IgG3 being the second-most abundant isotype (Figure 2D). Gene expression profiling also revealed differences between graft and blood B cell and PCs, as shown in Figure 2, E and F, and Supplemental Figure 2. Specifically, intragraft B cells expressed higher levels of 8 out of 10 genes characteristic of B1 B cell signatures in mice or innate-like B cells in humans (Figure 2F) (7, 8). In addition, expression of the *AHNAK* gene was upregulated in graft B cells and PCs compared with circulating cells, as was reported for innate-like B cells in rejected kidney transplants. Furthermore, gene set enrichment analysis (GSEA) identified a series of enriched pathways distinguishing infiltrating B cells and PCs combined from their blood counterparts (Supplemental Figure 3). In particular, several pathways associated with B cell proliferation and differentiation were upregulated in the graft B cells and plasmablasts (Pb)/PCs, including B cell receptor signaling, NF-κB signaling, several chemokine/cytokine signaling pathways, and apoptosis, suggestive of local B cell involvement in CAV through B cell differentiation into antibody-secreting plasma cells. Additionally, subcluster analysis of the Pb/PC population revealed 2 distinct clusters, one expressing early PC genes, found in both graft and PBMCs, and the other displaying a late PC gene expression signature, predominantly found in the graft (Supplemental Figure 4) (9, 10).

### Clonal composition of graft-infiltrating B cell subsets.

**Figure 3:**
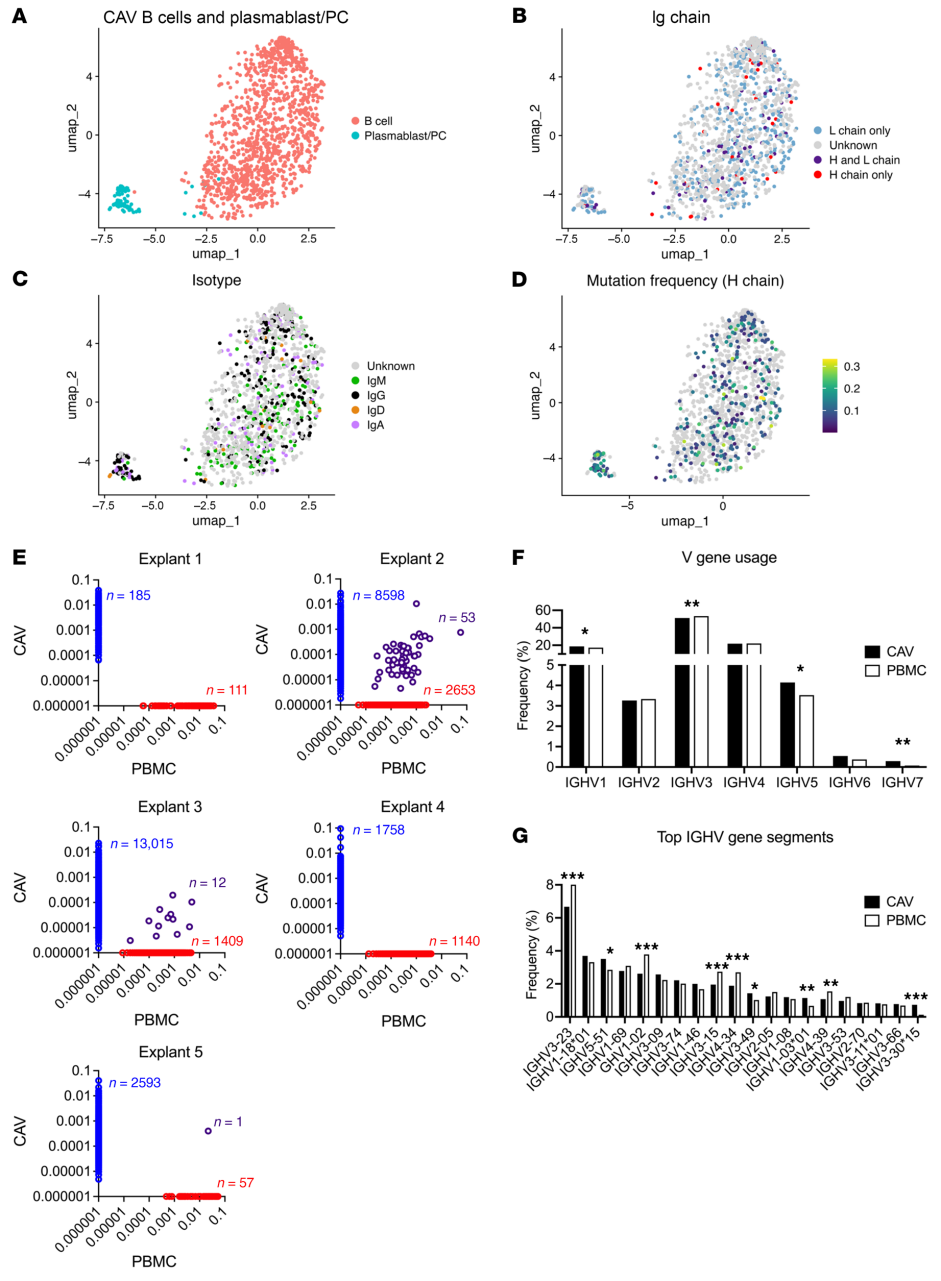
Clonal composition of graft-infiltrating B cells and plasmablasts/PCs in CAV. (**A**) UMAP embedding of intragraft B cells and plasmablast/plasma cells in CAV. (**B**) Expression of BCR heavy and light chains in CAV intragraft B cells and plasmablast/plasma cells. (**C**) Expression of immunoglobulin heavy chain isotypes IgM, IgG, IgD, and IgA in CAV B cells and plasmablast/plasma cells. (**D**) Frequency of complementarity-determining region 3 mutations in CAV intragraft B cells and plasmablast/plasma cells. (**E**) Comparison of shared BCR repertoires between CAV patient explants and autologous PBMCs. (**F**) Immunoglobulin heavy chain variable region gene usage of graft-infiltrating and circulating B cells and plasmablast/plasma cells. (**G**) The top 20 heavy chain variable gene segments between CAV and circulating B cells are shown. Statistical analyses were performed using Fisher’s exact test and *P* values depicted by *P < 0.05, **P < 0.01, ***P < 0.001.

To analyze the clonal composition of intragraft B cell and PC subsets, we carried out BCR profiling using the 5’ single cell V(D)J’ barcoded scRNAseq libraries generated from dissociated CD45^+^ cells from the 5 explants (Figure 3A). Productive paired immunoglobulin heavy and light chain rearrangements were obtained for B cells and PCs, as illustrated in Figure 3B. This analysis confirmed the predominance of IgG^+^ PCs within infiltrates (Figure 3C) and verified that virtually all PC clones had undergone somatic mutation (Figure 3D). To complement the BCR repertoire analysis, we sequenced rearranged IGHV genes using DNA extracted from a separate fragment of the 5 graft specimens used for scRNAseq/BCRseq as well as from the patients’ PBMC collected at time of retransplantation. As illustrated in Figure 3E, there was no or only minimal overlap between productive rearrangements found in the graft and those identified in the blood, corroborating our previous report that the clonotypic composition of infiltrating B cells was distinct from that of circulating B cells (5). The IGHV repertoires of intragraft and blood B cells were largely divergent. Analysis of immunoglobulin VH usage showed a predominance of rearrangements comprising IGHV3, IGHV4, or IGHV1 families in both graft and blood B cells (Figure 3F). Analysis of the top 20 sequenced gene segments revealed generally similar gene usage in both compartments. Moderate differences were observed with certain alleles elevated in the graft (IGHV5-51, IGHV3-49, IGHV1-03*01) while others were more prominent in blood B cells (IGHV3-23, IGHV1-02, IGHV3015, IGHV4-34, IGHV4-39) (Figure 3G). Moreover, we analyzed the clonal relationship between intragraft B cells, Pb/PC, and blood B cells. We considered BCR sequences sharing the same IGHV and IGHJ segment usage to be clonally related if they had the same CDR3 length and greater than 85% CDR3 sequence identity (11). Among 239 infiltrating PC and PBMC B cells sharing the same IGHV and IGHJ, only 1 exhibited a clonal relationship between blood and graft (Supplemental Table 2). In addition, among the 1,384 intragraft B cell and PBMC B cell BCR sequences with shared V-J usage, only 6 were clonally related. In contrast, within the graft compartment, 62 of 164 BCR sequences sharing V-J usage between intragraft B cells and PCs were also clonally related. While these results do not rule out the recruitment of fully differentiated PCs from the circulation into the graft, they suggest that a substantial proportion of intragraft PC differentiated in situ.

### Generation of recombinant monoclonal antibodies for intragraft PC.

To interrogate the antigen specificity of infiltrating PCs, we next generated recombinant monoclonal antibodies (mAb) as replicas of immunoglobulins produced in situ. To this aim, we isolated CD27^+^CD138^+^ PC clones expressing the tissue retention marker CD69 by single-cell sorting from cells dissociated from one of the 5 heart explants with CAV (explant 1). Additionally, CD27^+^CD138^+^ PCs were also isolated from PBMC from a healthy subject as controls. RNA extracted from individual PCs were then used as templates to clone paired immunoglobulin heavy and light chain cDNA in expression vectors and produce recombinant mAb. Nine mAb were generated from explant 1 and 24 mAb from a healthy participant using this method. The main characteristics of the 9 mAb derived from explant 1, including IGH gene segment usage and sequence comparison to germline, are listed in Table 1. The clone IGH CDR3 sequences are provided in Supplemental Table 1. To assess the abundance of the PC clones from which the 9 mAb were derived, we analyzed the frequency of their unique clonotypic CDR3 sequences in both scRNA-seq–derived V(D)J profiles and bulk repertoire sequencing data. Remarkably, none of the 9 clonotypic sequences could be found in the 2 datasets (Table 1), indicating that the corresponding PC clones were not among the most abundant clones in the graft infiltrate. As an alternate strategy to generate recombinant mAb, we used paired IGH/IGL sequences identified by V(D)J scRNAseq, focusing primarily on rearrangements detected in multiple copies and thus corresponding to PC clones expanded in situ. We selected 28 paired IGH/IGL sequences from the most dominant IgG1^+^ PC clones in the remaining 4 explants (explants 2–5) and generated recombinant monoclonal antibodies (mAb) by expression cloning. All 28 mAb were engineered as IgG1, matching the original antibodies secreted by the infiltrating PC, except for mAb9, which was secreted as IgG3 in the graft. The main characteristics of these 28 mAb, including IGH and IGL gene segments usage, are listed in Table 1, together with the frequency of the corresponding PC clones in the graft observed in both sRNAseq and bulk sequencing IGHV repertoire analyses. Of the 28 clonotypic sequences, 19 were detected either in multiple copies or in both scRNAseq and bulk IGHV repertoire datasets, indicating that they were derived from expanded PCs. The remaining 9 paired rearrangements were found at a single copy, although evidence of somatic mutations implied that the corresponding PC clones had undergone affinity maturation. The IGH and IGL CDR3 sequences are also reported in Supplemental Table 1.

### Reactivity profile of intragraft PC-derived mAb.

**Figure 4:**
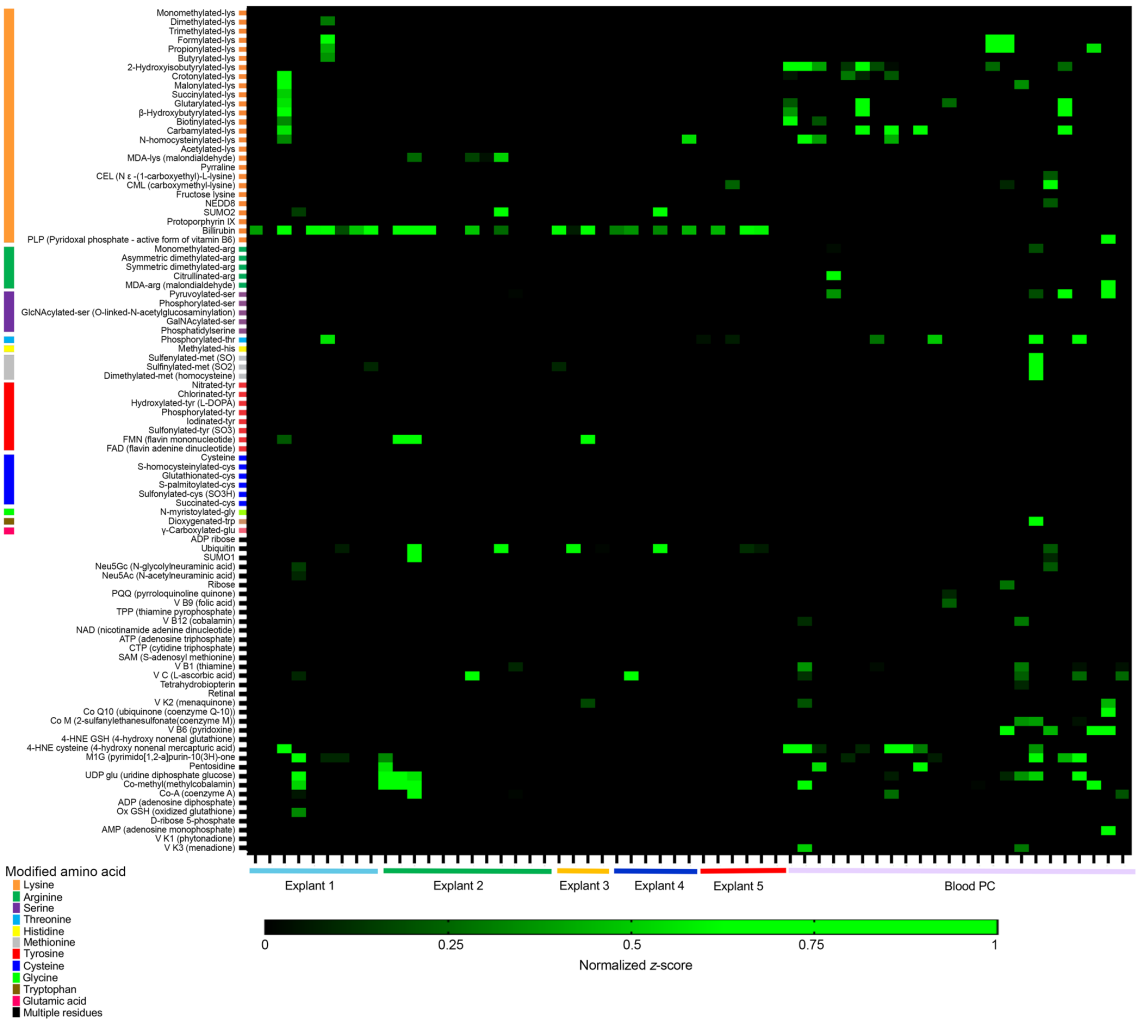
Reactivity profiles of graft-infiltrating PCs in CAV. Reactivity of 61 recombinant mab generated from 37 intragraft PCs obtained from 5 separate cardiac explants with CAV (left) and 24 peripheral blood PCs (right) to 93 common adducts. The source of the PC is indicated below the heatmap. The consensus target adduct, bilirubin, is recognized by a majority (21 of 37) of mab secreted by intragraft PC but none of the peripheral blood PCs. Reactivity to individual adducts is depicted as min-max normalized z-score values.

We first tested the reactivity of recombinant mAb to HLA, using complement-dependent cytotoxicity. None were reactive to HLA (Supplemental Figure 5). This lack of HLA reactivity in the mAbs generated from the infiltrating PCs was previously observed in immortalized graft-derived B cell clones. We previously reported that a majority of B cells found around coronary arteries with documented CAV lesions have a polyreactive profile characteristic of innate-like B cells (1). This initial observation aligned with the gene expression signature observed in our present study (Figure 2F). In addition, we recently uncovered that polyreactivity, the capacity of an antibody to bind multiple apparently unrelated antigens (i.e., different proteins, DNA), can often be explained by specificity to chemical adducts shared by the recognized antigens (6). Here, we reasoned that intragraft PCs may also target specific chemical moieties in the context of CAV. To test this hypothesis, we used a unique high-dimensional ELISA platform developed in our lab to assess the reactivity of the 37 mAb generated from intragraft PC clones as well as the 24 control mAb derived from peripheral blood of a healthy donor to 93 chemical adducts. Our panel comprises 60 posttranslational modifications and 33 metabolites and cofactors binding covalently or noncovalently to proteins or other macromolecules. A list of all 93 adducts is provided in Supplemental Table 3. As shown in Figure 4, a majority of mAbs generated from graft PC (21 of 37) recognized bilirubin, a degradation product of heme catabolism affixed to lysine residues. In contrast, the 24 mAb derived from healthy blood PCs reacted to multiple adducts but not bilirubin. These results indicated that bilirubin was a dominant target of local PC responses associated with CAV. As additional controls, we generated mAb from the blood of 2 of the 5 patients for whom intragraft PC were characterized (patients 2 and 3). As no CD138^+^ PCs could be detected in the patient PBMCs, we derived recombinant mAb from single cell–sorted memory CD20^+^ CD27^+^ memory B cell clones. A total of 96 (*N* = 61 for patient 2 and *N* = 35 for patient 3) were generated and tested for their reactivity to bilirubin. Only 1 of 96 mAb reacted to this metabolite, indicating that the frequency of B cells with this specificity in PBMC is low (Supplemental Figure 6).

### Bilirubin deposition in graft infiltrates in CAV.

**Figure 5:**
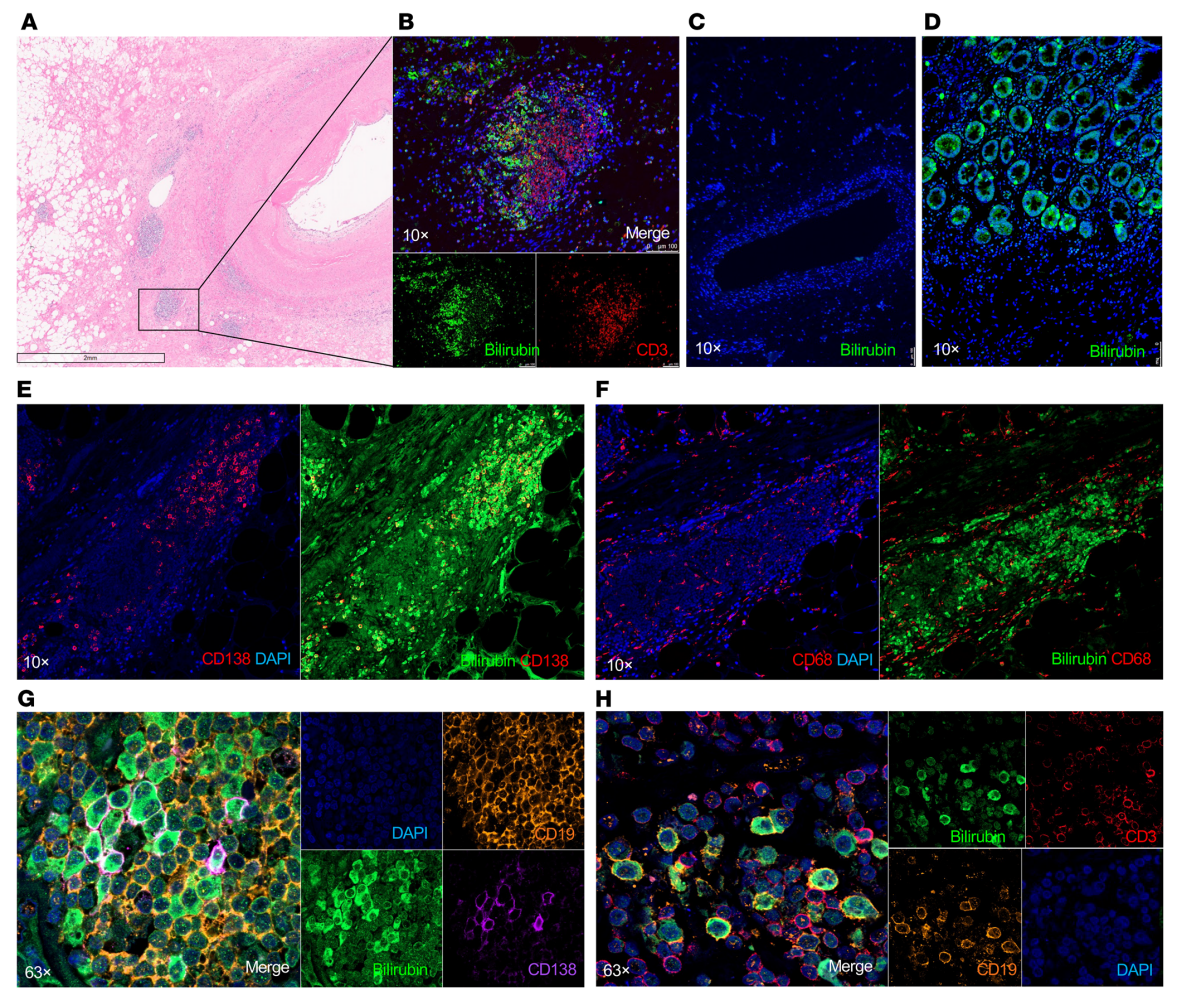
Bilirubin accumulates in immune graft-infiltrates in CAV. (**A**) H&E staining of a representative heart explant with CAV, showing dense immune infiltrates around and in the adventitia of a coronary artery showing evidence of intimal hyperplasia. (**B**) Bilirubin, CD3, and DAPI staining of the same explanted allograft with CAV section shown in **A**. Bilirubin staining is observed in lymphoid aggregates. (**C**) Bilirubin and DAPI staining of healthy heart tissue. (**D**) Bilirubin and DAPI staining of obstructed bile ducts. (**E**) Bilirubin, CD138 and DAPI staining of a representative lymphoid aggregate in an explanted allograft with CAV. (**F**) Bilirubin, CD68, and DAPI staining of a lymphoid aggregate in an explanted allograft with CAV. (**G**) Bilirubin, CD19, CD138, and DAPI staining of a lymphoid aggregate in an explanted allograft with CAV. (**H**) Bilirubin, CD3, CD19, and DAPI staining of a lymphoid aggregate in an explanted allograft with CAV Images were obtained with a Leica DMI 6000 microscope or a Zeiss LSM 900 confocal microscope. Magnification, ×10 (**B**, **C**, **D**, **E**, and **F**) or ×63 (**G** and **H**). Scale bars: 2 mm (**A**); 100 μm (**B**).

**Figure 6:**
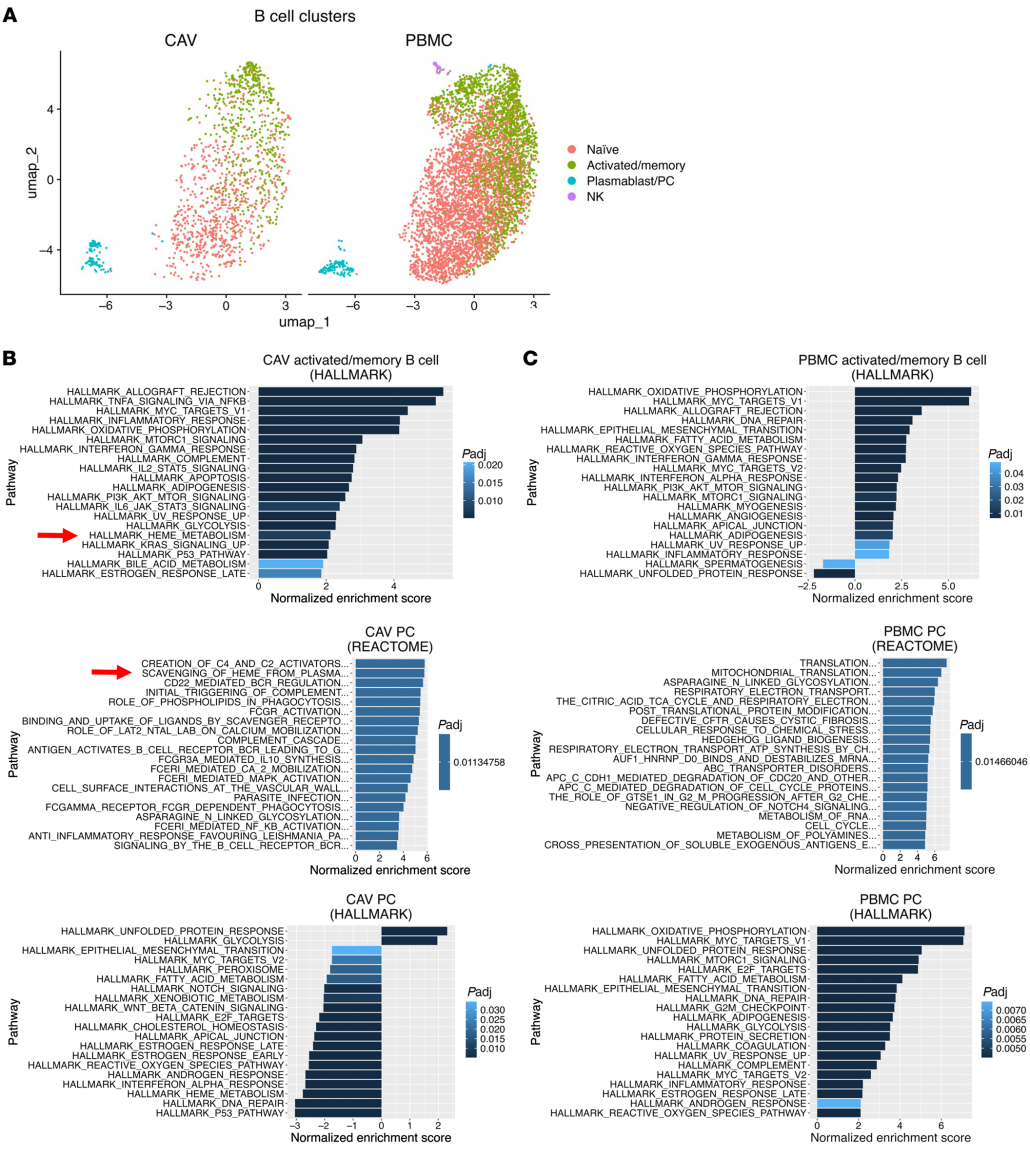
Bilirubin-related (heme) pathways are enriched in B cell subsets in CAV. (**A**) UMAP embeddings of B cell subsets in CAV and healthy donor peripheral blood mononuclear cells. (**B** and **C**) Results of gene set enrichment analysis of ranked differentially expressed genes in B cell subsets (activated/memory B cells or PC) in CAV and healthy donor PBMC revealed enrichment of heme-related pathways in human Hallmark and Reactome gene sets in CAV.

The observation that infiltrating PCs, including dominant clones, reacted to bilirubin in 5 out of 5 explants with CAV, suggested that accumulation of this metabolite in situ could have triggered these local antibody responses. To test this proposition, we stained graft tissue surrounding diseased coronaries with evidence of intimal hyperplasia for the presence of bilirubin. Intense staining was detected in immune infiltrates in graft tissue collected from 16 out of 16 heart transplant recipients with CAV, including the 5 cases we analyzed by scRNAseq (Supplemental Table 4). In contrast, no bilirubin could be detected in healthy heart autopsy tissue from 5 participants who died from noncardiac causes as well as cardiac grafts without CAV (Supplemental Figure 7). An example of bilirubin staining of a representative immune infiltrate is depicted in Figure 5, A and B, together with control healthy heart tissue (Figure 5C) and positive control tissue from obstructed bile ducts (Figure 5D). Bilirubin appeared to be deposited unevenly in areas showing dense infiltrates of CD138^+^ PCs (Example in Figure 5E) but not CD68^+^ macrophages (Figure 5F). Higher magnification revealed nuclear dots of bilirubin in virtually all immune cells; however, homogenous cytoplasm staining for this metabolite was observed in only 2 cell types: CD19^+^ B cells and CD138^+^ PCs (Figure 5, G and H). Notably, only a fraction of B cells and PCs stained positively for bilirubin in the cytoplasm. This exclusive cytoplasm accumulation of bilirubin in a fraction of B cells and PCs concurred with their gene expression signature. As depicted in Figure 6, gene set enrichment analysis identified “heme metabolism” as one of the top 15 gene sets from the Hallmark molecular signatures dataset in activated/memory B cells from grafts with CAV (Figure 6, A and B) but not in blood activated/memory B cells (Figure 6, A and C). Likewise, “scavenging of heme from plasma” was the second-top gene set from the Reactome dataset identified in graft PCs (Figure 6B) but not circulating PCs (Figure 6C). Genes involved in heme degradation were expressed in CAV B cells and included BLVRA, BLVRB, HMOX1, HMOX2, ABCC1, and GUS1.

To assess the presence of bilirubin deposits prior to CAV, we also examined 16 serial clinical biopsies collected up to 20 years before graft explantation from patient 2. Immunofluorescence staining showed the presence of immune infiltrates, including for CD19^+^ B cells, CD138^+^ plasma cells, CD3^+^ T cells, as well as bilirubin deposits in 4 of 16 biopsies at different time points posttransplant and before retransplantation (Supplemental Table 5 and Supplemental Figure 8). These biopsies were classified as ACR ISHLT 0, 1R, or 2R.

### Bilirubin deposition in the tunica media of coronary arteries in CAV.

**Figure 7:**
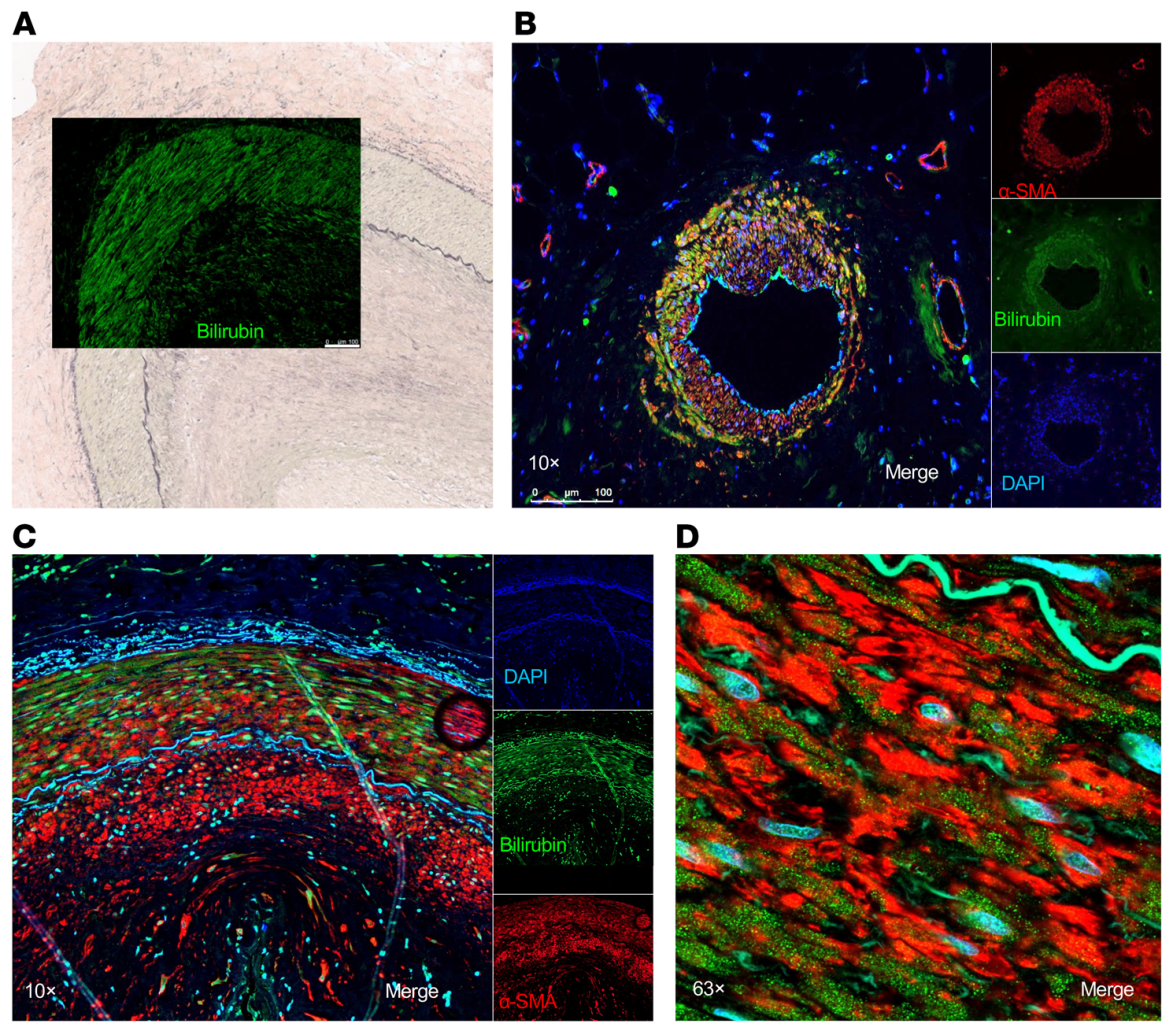
Bilirubin deposition is observed primarily in the tunica media of coronary arteries with CAV. (**A**) Superimposed pictures of immunofluorescence staining for bilirubin (green) and Van Gieson elastin staining of a transplanted heart tissue with CAV. (**B**–**D**) Immunofluorescence staining for bilirubin (green) and α-smooth muscle cell actin (α-SMA, red) in cardiac tissue with CAV. Images were obtained with a Leica DMI 6000 microscope or a Zeiss LSM 900 confocal microscope, magnification, × 10 (**B** and **C**) or × 63 (**D**). Scale bars: 100 μm (**A** and **B**).

In addition to immune infiltrates, intense bilirubin staining was also observed in transplant coronary arteries with evidence of intimal hyperplasia, as illustrated in Figure 7A. Bilirubin accumulated primarily in smooth muscle cells of the tunica media but was also found, to a lesser extent, in the intima layer where these cells migrated. Such bilirubin deposition in coronary arteries was observed in 8 out of the 16 CAV cases we examined but none of the 5 control healthy heart tissue. As shown in Figure 7, B–D, bilirubin was detected as dots in both the cytoplasm and nuclei of smooth muscle cells identified by α-smooth muscle actin.

### MAbs derived from intragraft PCs react to explant tissue with CAV.

**Figure 8:**
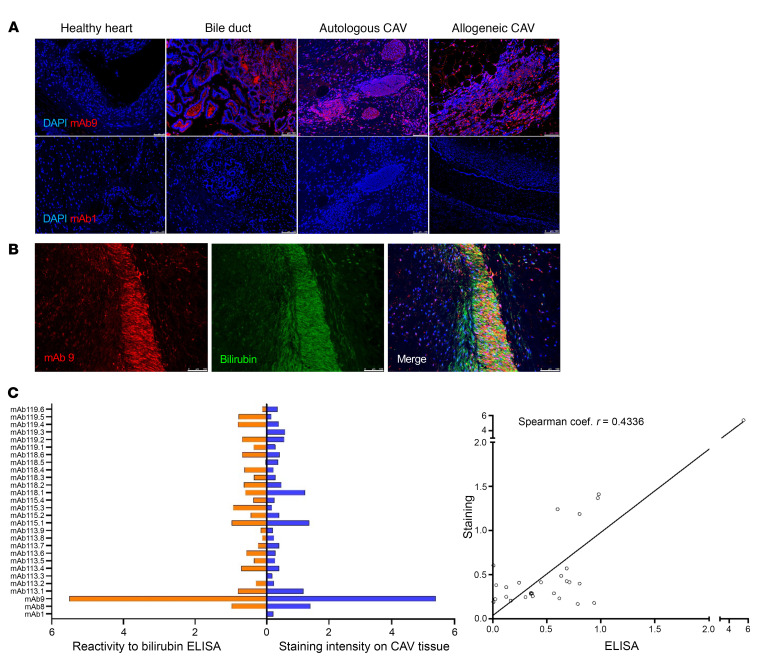
Monoclonal antibodies derived from intragraft PCs react to cardiac tissue with CAV. (**A**) Immunofluorescence staining of tissue obtained from healthy heart, obstructed bile ducts, autologous and allogeneic heart explants with CAV with a representative bilirubin-reactive mAb (mAb9) and non-reactive mAb (mAb1). Both mAb were generated from a graft-infiltrating PC expanded in situ and reactive to bilirubin. (**B**) Immunofluorescence co-staining of tissue from a heart transplant with CAV with a representative bilirubin-reactive mAb (mAb9) as well as anti-bilirubin antibodies. (**C**) Correlation between mAb reactivity to bilirubin assessed by ELISA and reactivity to heart explant with CAV assessed by immunofluorescence (*N* = 28). The spearman rank correlation coefficient was calculated after MFI and ELISA data were z-transformed to ensure scale-independent comparability (*r* = 0.4336, *P* = 0.0211). Images were obtained with a Leica DMI 6000 microscope, magnification, × 10. Scale bars: 75 μm (**A**, left-most image); 100 μm.

We next assessed the capacity of the 28 mAbs engineered from the IGH/L sequences of dominant infiltrating PCs to directly bind cardiac tissue from patients with CAV. Figure 8A shows the staining of a mAb highly reactive to bilirubin by ELISA (mAb9) as well as one that did not react (mAb1) to control healthy heart, obstructed bile ducts, autologous tissue, i.e., tissue obtained from the same patient from whom the mAb was derived, as well as allogeneic tissue i.e., tissue from a different patient. As expected, mAb9 reacted to autologous and allogeneic cardiac tissue with CAV as well as bile duct tissue, but not that of healthy heart devoid of bilirubin. These results show that antibilirubin mAb react to this metabolite in graft tissue regardless of the source of the specimen. Staining of all remaining mAbs is reported in Supplemental Figure 9. MAb9 also recognized bilirubin deposited in the coronary artery media (Figure 8B). Overall, we observed a moderate correspondence between reactivity to bilirubin measured by ELISA and staining of CAV explant tissue (Figure 8C).

### CAV associates with heme degradation in coronary arterial walls.

**Figure 9:**
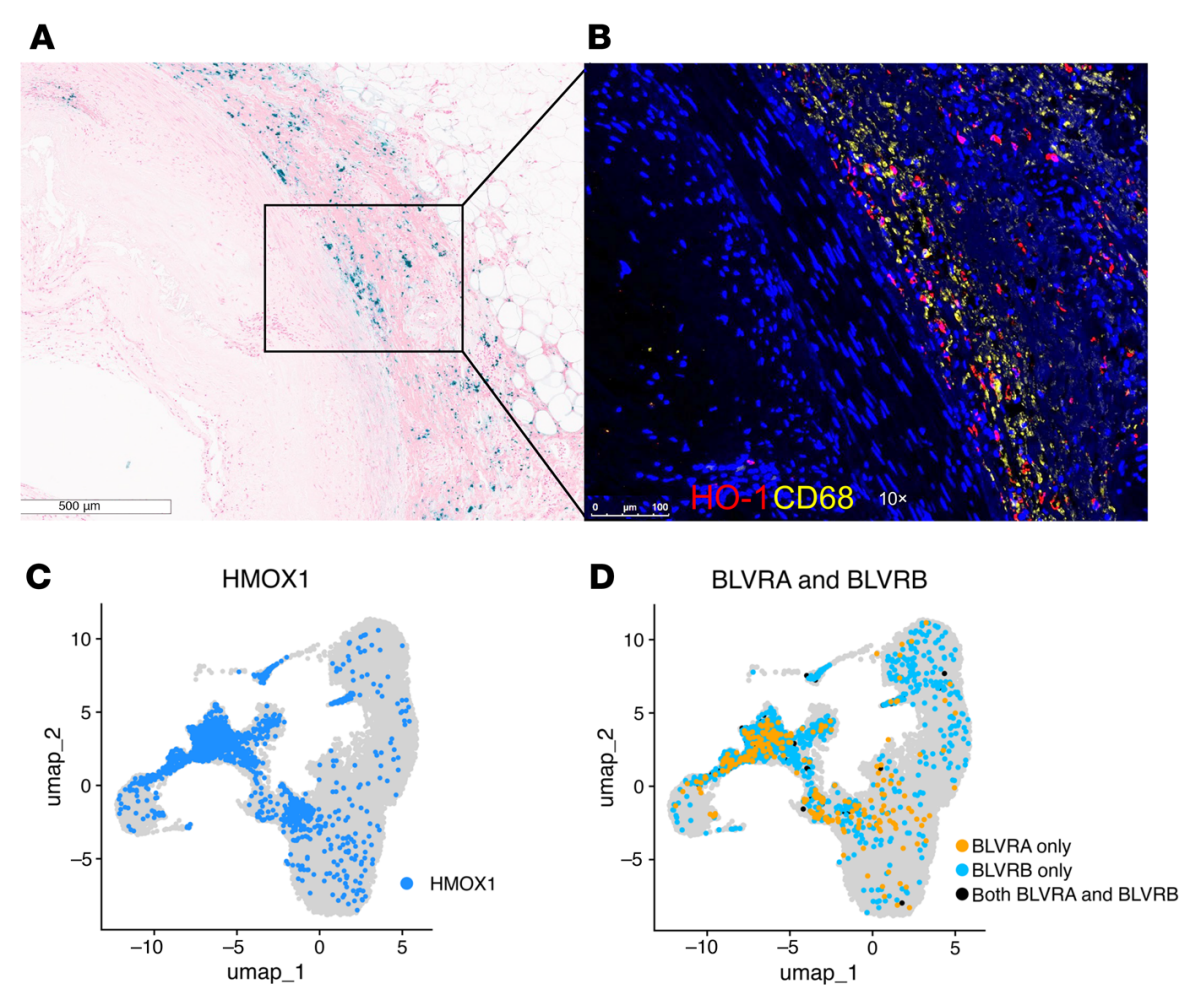
Heme degradation is prominent in the tunica media of coronary arteries with CAV. (**A**) Prussian blue staining of heart transplant tissue with CAV showing Fe^2+^ iron deposits in an affected artery. (**B**) Immunofluorescence staining for HO-1 (red) and CD68 (yellow) in a heart transplant artery with CAV lesion. (**C** and **D**) ScRNA-seq–integrated analysis of CD45^+^ cells isolated from 5 explanted cardiac grafts showing high expression of HMOX1, BLVRA, and BLVRB genes by infiltrating macrophages. Images were obtained with a Leica DMI 6000 microscope; magnification, ×10. Scale bar: 100 μm.

The abundance of bilirubin in the graft suggested that this metabolite was produced through heme catabolism in situ during CAV. To verify this hypothesis, we used Prussian blue staining to test for the presence of ferrous ion, Fe^2+^, another degradation product of heme, in cardiac graft tissue with CAV. As shown in Figure 9A and Supplemental Figure 10, Fe^2+^ was readily detectable in the tunica media of coronary arteries with evidence of intimal thickening. Fe^2+^ was found in areas richly infiltrated with CD68^+^ macrophages where heme oxygenase–1 (HO-1), the main enzyme responsible for breaking down heme was also detected (Figure 9B). ScRNAseq analysis confirmed robust expression of HO-1 gene (HMOX-1) as well as biliverdin reductase A and B genes (BLVRA and BLVRB), predominantly in graft-infiltrating macrophages, but in other graft cells too (Figure 9, C and D).

### Serum levels of anti-bilirubin IgG decrease in heart transplant recipients with advanced CAV.

**Figure 10:**
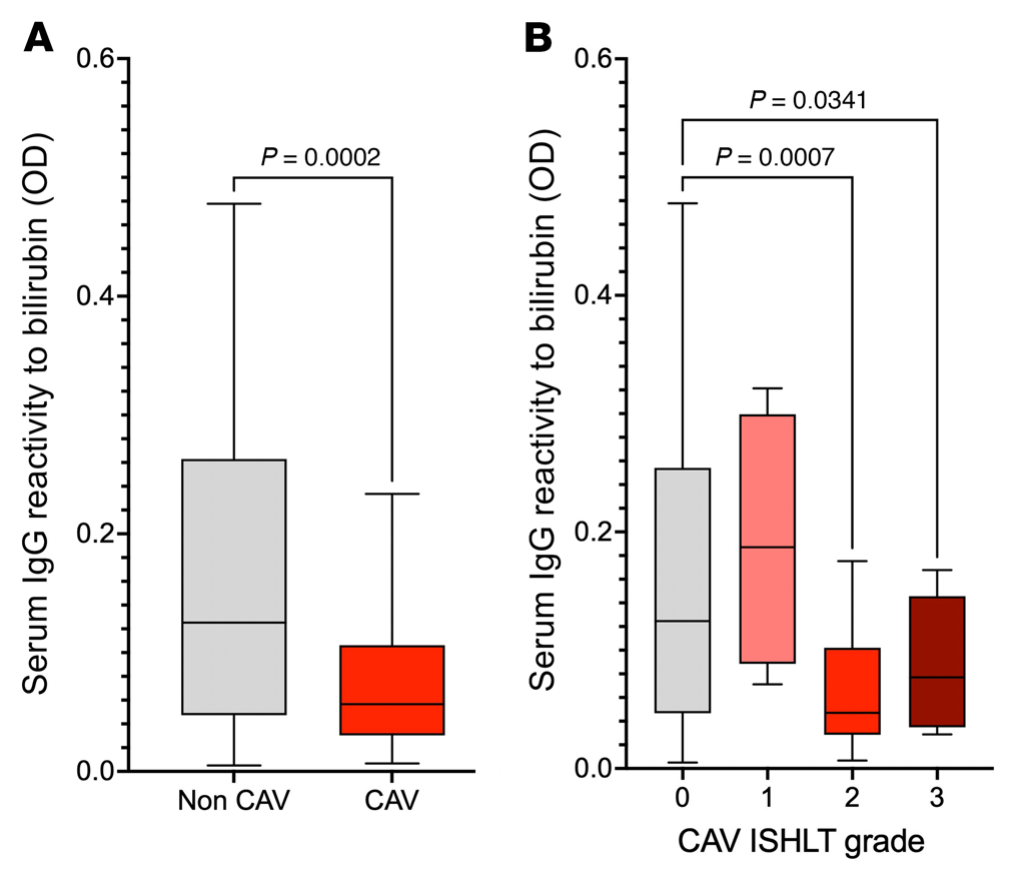
Serum levels of anti-bilirubin IgG are decreased in heart transplant recipients with advanced CAV. (**A**) Serum IgG reactivity to bilirubin assessed posttransplant in heart transplant recipients with (*N* = 44) or without CAV (*N* = 40). The *P* value was calculated using Welch *t* test. (**B**) Posttransplant serum IgG reactivity to bilirubin in heart transplant recipients with CAV ISHLT score 1 (*N* = 4), score 2 (*N* = 26), score 3 (*N* = 14) or without CAV (*N* = 40). Serum specimens were diluted 1:1,000 before testing. *P* values were calculated using the Dunnett’s T3 multiple comparisons test.

While bilirubin was deposited in the transplanted heart during CAV, this was not accompanied by hyperbilirubinemia. The 16 heart transplant recipients whose explants showed bilirubin deposition had total bilirubin levels within normal range (mean 1.14 mg/dL). In previous studies, we reported on the development during early life and the maintenance across the lifespan of humoral immunity to a broad spectrum of chemical adducts, including bilirubin (6). Here, we tested serum levels of anti-bilirubin IgG in heart transplant recipients with CAV (*N* = 44) or without this complication (*N* = 40) treated at 2 separate institutions, the Bellvitge University Hospital and the Columbia University Medical Center. The main characteristics of patients in the 2 cohorts are listed in Supplemental Table 6. As depicted in Figure 10A, patients with CAV showed reduced levels of circulating anti-bilirubin IgG compared with patients without this form of rejection. Moreover, serum anti-bilirubin IgG levels were only decreased in transplant recipients with a moderate to severe form of CAV (ISHLT grade 2 to 3, Figure 10B). No difference in total bilirubin levels was observed between CAV and non-CAV groups in the 2 cohorts (Supplemental Figure 11). Lastly, to investigate whether decreased IgG levels was due to adsorption of circulating anti-bilirubin antibodies in the graft, we eluted IgG from cardiac tissue with CAV (*N* = 5) and from control non-CAV tissue (*N* = 5) and tested their reactivity to bilirubin. Overall, IgG eluted from CAV tissue were less reactive to bilirubin than IgG eluted from control healthy heart tissue, matching the results obtained with serum samples (Supplemental Figure 12).

## Discussion

PC infiltrates have been associated with different types of rejection in both experimental models and human transplants. Early studies by the Lakkis group described intragraft PC as part of local immune responses in heterotopic allogeneic heart transplants during CAV (12). Thaunat et al. further analyzed immune infiltrates in human kidney grafts and proposed that PC are generated in situ in structures reminiscent of germinal centers called tertiary lymphoid organs (TLO) (13). Despite their abundance in allografts and their presumed contribution to the rejection process, only limited information is available about the antigen specificity of infiltrating PC. A recent study examined the reactivity of antibodies generated from B cells in kidney biopsies and found that a majority were reactive to self antigens (14). However, only a few of these antibodies were derived from differentiated PCs. In contrast, PCs infiltrating human heart transplant during CAV had not been characterized until now. Here, we used a strategy combining scRNAseq, immune receptor repertoire profiling, monoclonal antibody engineering, and a unique high-dimensional ELISA platform to identify the reactivity of infiltrating PCs with a focus on dominant clones in situ more likely to have contributed to the rejection process.

Our scRNAseq analysis revealed 4 major types of immune cells in graft infiltrates during CAV: T cells, NK cells, macrophages, and B cells/PC. Our analysis found a gene signature characteristic of innate-like B cells. This observation is consistent with our prior study reporting the prevalence of intragraft innate-like B cell clones in human CAV (1). Moreover, our findings also corroborate the previous description of innate-like self-reactive B cells infiltrating rejected kidney allografts by Asano and colleagues (14). Collectively, these studies conducted in 2 different organs support the general view that B cells involved in local immune responses during human transplant rejection display characteristics of innate B cells, also called B1 B cells, in mice (15). Of note, these cells are distinct from follicular B cells responsible for DSA production.

As we previously reported, there was evidence of clonal dominance among infiltrating B cells with minimal overlap between blood and graft IGHV repertoires (5). Additionally, through scRNAseq analysis, we demonstrated the presence of dominant PC clones in all 5 explants. Such clonal dominance likely resulted from antigen-driven proliferation and expansion of specific clones in situ. It is also possible, albeit less probable, that PC clones with certain specificities were preferentially recruited, explaining their enrichment in the graft compared with the blood. Nevertheless, in both scenarios, the most abundant clones either expanded at the site or recruited “en masse” are likely to be dominant players in the local immune responses associated with CAV.

Our analysis also revealed the predominance of IgG1- followed by IgG3 and IgA1-producing cells among infiltrating PC. This observation is in keeping with our previous finding that antibodies secreted by graft infiltrates surrounding the coronary arteries in CAV are predominantly of the IgG isotype (1). Additionally, with respect to the preferential IgG isotype profile, we have previously reported on the bias toward IgG1 and IgG3 of antibodies reactive to apoptotic cells or malondialdehyde, a prominent oxidation epitope (16, 17). Antibodies with such specificity are commonly secreted by innate-like B cells comparable with those found in the graft during CAV.

Furthermore, IgG1 and IgG3 are complement activating Ig subclasses likely involved in the pathophysiology of CAV. In contrast, the function of IgA1 in this complication is entirely unknown. Recent advances in cancer immunology suggest a critical role of IgA1 in sensitizing tumor cells following transcytosis mediated by the polymeric immunoglobulin receptor pIgR (18, 19). Whether a comparable mechanism is unfolding in the context of transplant rejection is unclear.

The identification of bilirubin as a predominant target of intragraft PC in CAV was unexpected. Additional adducts were also recognized, yet bilirubin was unquestionably the consensus target of a majority of clones derived from 5 of 5 explants. None of the 24 control mAb derived from PCs isolated from healthy donor PBMC reacted to bilirubin, indicating that the frequency of circulating PC clones with this specificity is low at the steady state. This clearly contrasts with the enrichment of bilirubin-reacting clones within graft infiltrates, which, again, points to either their expansion in situ or their preferred recruitment from the blood. We recently reported on the development and maintenance of a broad repertoire of anti-adduct antibodies across the human lifespan (6). Our study showed that anti-bilirubin humoral immunity develops during the first year after birth and is maintained through life. Such serum reactivity implies the existence of bilirubin-specific memory B cells and PCs at the steady-state, even if their frequency in the peripheral blood may be low. In light of these previous findings, our new data suggest that B cell responses to bilirubin observed in heart grafts with CAV did not arise de novo but were instead derived from a preexisting natural immunity to this metabolite.

Bilirubin is one of 3 end products of heme catabolism together with carbon monoxide and Fe^2+^ (20). The source of heme contributing to bilirubin deposition in the graft is likely dual: hemoglobin from broken down erythrocytes in perivascular immune infiltrates and hemoproteins in smooth muscle cells in the tunica media. Poorly soluble as a free metabolite, bilirubin is known to bind albumin in the serum (21). How it accumulates in tissue in general, and transplanted organs, in particular, is less understood. Pioneering studies conducted in experimental models by 2 separate investigative teams uncovered a protective role of HO-1, the heme degradation pathway and bilirubin in-graft rejection (22–24). A comparable role could be envisioned in human transplants. On the other hand, at high levels, the deposition of bilirubin in tissue can become toxic as exemplified by neurological damage associated with kernicterus, a condition caused by hyperbilirubinemia in newborns (25). Irrespective of its biological role, it is likely that bilirubin accumulation in the graft triggered B cell responses, leading to the differentiation of clones specific to that metabolite into antibody-producing cells. This mechanism would be analogous to the development of antibodies to modified self structures in several autoimmune diseases, such as the generation of anticitrullinated protein antibodies (ACPA) in rheumatoid arthritis (26). However, it is surprising, that, in the context of HLA-mismatched allogeneic heart transplantation, the major component of local B cell immunity be directed towards modified self determinants rather than donor-specific HLA. An important limitation to our study is that we did not establish the exact role and contribution of anti-bilirubin antibodies to the pathophysiology of CAV. Drawing a parallel with ACPA in RA, these bilirubin-reactive antibodies could form immune complexes with proinflammatory properties, exacerbating local immune reactions. These antibodies could also participate in the clearance of bilirubin from the graft, thereby counteracting its immunoregulatory function.

Our experiments also revealed a distinctive staining pattern for bilirubin in graft infiltrates. While most immune cells displayed dots of bilirubin in the nucleus, B cells and PCs also showed its accumulation in the cytoplasm. However, only a fraction of these cells stained positively for bilirubin in the cytoplasm. An intriguing possibility is that cells showing bilirubin in the cytoplasm may express a BCR specific to bilirubin, allowing them to recognize and internalize this metabolite conjugated to albumin or other proteins. Further research will verify this hypothesis.

Lastly, we observed decreased serum levels of anti-bilirubin IgG in patients with advanced CAV, (ISHLT grade 2 and 3) compared with patients with only mild CAV or without this complication (ISHLT 0 or 1). This difference would suggest adsorption of the corresponding antibodies in the graft. However, the evidence does not support this possibility. First, most bilirubin accumulation in the graft appears to be intracellular, localizing either in nuclei or in the cytoplasm of B cells, plasma cells, and smooth muscle cells, and is therefore unlikely to result in antibody adsorption. Second, IgG eluted from cardiac tissue with CAV showed less reactivity to bilirubin when compared with those eluted from control heart tissue without the complication arguing against the adsorption hypothesis. A more plausible explanation is that circulating anti-bilirubin IgG, together with intragraft PC–derived antibodies, bind free bilirubin and facilitate its clearance.

In conclusion, the unexpected identification of bilirubin as a prominent antigenic structure targeted by dominant intragraft PC casts a new light on local B cell responses in human CAV. Our findings also highlighted heme catabolism as a pivotal feature of CAV, suggesting that strategies to influence this pathway may mitigate the severity of the disease.

## Methods

### Sex as a biological variable.

Our study included both male and female cardiac transplant recipients, and similar findings are reported for both sexes.

### Patients.

Cardiac graft surgical specimens and peripheral blood samples from 5 heart transplant recipients (explants 1–5) with cardiac allograft vasculopathy (ISHLT CAV2 or CAV3) who were undergoing a second heart transplantation procedure between 2020 and 2021 at the Columbia University Irving Medical Center (CUIMC) and Cedars Sinai Medical Center (CSMC) were used for scRNAseq experiments. Formalin-fixed paraffin-embedded (FFPE) cardiac explant tissue obtained from an additional 11 patients with CAV who underwent retransplantation at CUIMC (explants 6–16) were also used for IHC. Characteristics of these 16 patients are detailed in Table 2 and Supplemental Table 4. Sixteen FFPE biopsy sections collected from patient 2 through a 20-year period preceding retransplantation were also assessed by immunofluorescence (Details in Supplemental Table 5). Control specimens included FFPE tissue fragments for cardiac graft without CAV (*N* = 5) as well as healthy heart tissue obtained from autopsy. In addition, serum specimens collected from heart transplant recipients with or without CAV treated at the Bellvitge University Hospital (Barcelona, Spain; *N* = 31) and CUIMC (*N* = 53) were used in this study. The main characteristics of the patients included in the 2 cohorts are listed in Supplemental Table 6.

### Cardiac explant collection and processing.

Tissue fragments were obtained from different locations of the explanted allograft corresponding to the area immediately surrounding the left anterior descending coronary artery, right coronary artery, and circumflex coronary artery. Samples from the proximal, mid, and distal regions of each coronary artery were collected and stored unfixed in RPMI for up to 72 hours at 4°C until processing.

### Sample processing and dissociation.

In brief, coronary-adjacent tissue was minced, digested with collagenase and hyaluronidase, dissociated with an automated tissue dissociator (Miltenyi Biotech), and strained through a 100 μm cell strainer. Myocytes, adipose, and cell debris were separated from the strained cells by density-gradient centrifugation and discarded. The remaining cells were used for scRNA library preparation and sequencing. Additional cardiac graft fragments were either flash frozen for immunoglobulin heavy chain repertoire analysis or fixed in 10% formalin prior to paraffin embedding. Peripheral blood mononuclear cells were isolated from fresh peripheral whole blood taken at the time of retransplant by density-gradient centrifugation.

### Droplet-based scRNAseq.

Cells from dissociated explanted cardiac grafts were stained with Aqua dead cell dye (L34957, Invitrogen) and BV421 anti-CD45 antibody (368521, Biolegend). All viable cells as well as viable CD45^+^ cells were sorted on an Influx cell sorter (BD Biosciences) and processed for 10X Genomics scRNAseq by generating Chromium Next GEM single cell 5′ gene expression libraries and single cell V(D)J expression libraries, respectively. Sequencing was performed on an Illumina NovaSeq platform and single-cell sequencing reads were processed and aligned to the human reference genome GRCh38-5.0.0 using the CellRanger v5.0.1 or v6.1.2 or Cell Ranger V(D)J v6.1.1 or v6.1.2 pipelines. Quality control, filtering, and normalization of raw data was followed by integration, dimensional reduction, clustering, and differential gene expression analysis and were performed using the Seurat v5.3.0 package for R. For PBMC scRNAseq data, publicly available data from healthy donors were accessed, integrated, and analyzed using the same methods as for the CAV data: PBMC_68K dataset (27) (DOI: 10.1038/ncomms14049, Sequence Read Archive (SRA) accession SRP073767); PBMC_10K dataset (https://support.10xgenomics.com/single-cell-gene-expression/datasets/3.0.0/pbmc_10k_v3); PBMC Amit dataset (28) (DOI: 10.1016/j.cell.2020.06.032, Gene Expression Omnibus (GEO) accession GSM4560070). Cell-type identities were inferred using the SingleR v2.4.0 and Azimuth v0.5.0 R packages and confirmed using the EasyCellType v1.4.0 package for R and by comparing differentially expressed markers obtained using the FindAllMarkers and FindMarkers functions in the Seurat R package. Inference of pseudotemporal trajectories were performed using the Monocle 3 v1.3.7 R package.

### Immunoglobulin heavy chain repertoire analysis.

Genomic DNA from flash frozen explanted cardiac tissue and autologous peripheral blood mononuclear cells from four patients (explants 1–4) were extracted with DNeasy Blood and Tissue kit (Qiagen, Hilden, Germany). High-throughput, survey-level immunoglobulin heavy chain variable region sequencing was performed by Adaptive Biotechnology. CDR3 sequences from autologous cardiac tissue and PBMC were compared for sequences present in both tissue locations.

### Single cell mAb cloning, sequencing, and expression.

Heavy and light chain sequences were obtained from scRNA-seq V(D)J expression sequencing of CD45^+^ CD138^+^ or MZB1^+^ cells from four patients (explants 2–5) and were submitted to Genscript for the generation of recombinant monoclonal antibodies. Briefly, genes were synthesized and cloned into expression vectors containing human IgG1 backbone and either human IgK or IgL. Transient transfection into Chinese hamster ovary cells optimized for high-throughput antibody production was performed, followed by protein A purification. For one patient (explant 1) mAb were generated as previously described (7). Briefly, single cells dissociated from explanted heart tissue were stained with Aqua dead cell dye (L34957, Invitrogen), PE-Dazzle 594 anti-CD3 (300450, Biolegend), APC-Cy7 anti-CD27 (25-0279-T100, Tonbo), Alexa Fluor 700 anti-CD14 (557923, BD Biosciences), APC anti-CD19 (Biolegend, 302212), PE anti-CD138 (130-119-840, Miltenyi Biotec), FITC anti-CD20 (35-0209-T100, Tonbo), PerCP-Cy5.5 anti-CD69 (310925, Biolegend), BV711 anti-CD38 (563965, BD Biosciences) and BV421 anti-CD45 (368521, Biolegend). Viable CD45^+^ CD27^+^ CD69^+^ CD138^+^ cells were sorted into hypotonic lysis buffer in 384-well plates and stored at –80°. cDNA was generated from mRNA isolated from single B cells. PCR IgVH and IgVK/L amplicons, generated from the single cell cDNA, were cloned into expression vectors containing the constant regions of human IgG1, IgK, or IgL. Plasmids were subjected to Sanger sequencing. Pilot expression was performed by cotransfecting 1 μg of IgH and IgL plasmids into 1 mL of 293FreeStyle cell culture (Invitrogen) using polyethyleneimine (PolySciences). Following 3 days in culture, conditioned media was harvested and cleared by centrifugation. The same method was used to generate mAb from single-cell sorted CD19^+^CD27^+^ memory B cell clones from patients 2 (*N* = 61) and 3 (*N* = 35).

### Adduct ELISA detection of mAb reactivity to chemical adducts.

MAbs were tested for reactivity to a panel of 93 adducts using a modified ELISA platform based on a previously established method (6). The ELISA panel consisted of the following components: (a) 46 synthetic peptides, each 5 amino acids long, featuring a chemically modified residue flanked by 2 unmodified arginine on either side (control peptides with unmodified residues were also included); (b) 6 chemically altered amino acids; (c) 8 posttranslational modification (PTM) compounds, such as SUMO and ubiquitin; and (d) 33 cofactors, coenzymes, and metabolites. MDA-modified BSA and MDA-linked lysine/arginine peptides were generated by incubating acid-hydrolyzed 1,1,3,3-tetramethoxypropane (Sigma-Aldrich) with BSA or lysine/arginine peptides. In summary, 2 M 1,1,3,3-tetramethoxypropane was hydrolyzed in 96 mM HCl at 37°C for 15 minutes, then neutralized with NaOH. BSA (2 mg/mL) or lysine/arginine peptides (10 μg/mL) were incubated with 0.2 M MDA at 37°C for 3 hours, followed by extensive dialysis against 1X phosphate-buffered saline (PBS) at 4°C for 36 hours. A comprehensive list of adducts and controls, including their sources and working concentrations, is available in Supplemental Table 3. MAb reactivity to adducts were tested in duplicate. Briefly, small compounds, synthetic peptides with modified amino acids, and large posttranslational modification compounds were coated onto ELISA plates at 1 mM, 10 μM, and 1 μM, respectively. Plates were washed and blocked with 3% BSA followed by incubation with mAbs for 3 hours at room temperature. IgG was detected with anti-human IgG F(ab’) secondary antibody (709-036-098, Jackson ImmunoResearch Laboratories Inc.) for 1 hour at room temperature and developed with 3,3′,5,5′-tetramethylbenzidine (TMB, Fisher Scientific Inc.).

### Serum IgG reactivity to bilirubin.

Serum IgG reactivity to bilirubin was assessed by ELISA. In brief, Corning Clear Polystyrene 96-Well EIA/RIA Microplates were coated in duplicate with either bilirubin (Cayman Chemicals) at a final concentration of 1 mM or DMSO as controls and incubated for 20 hours at 4°C. The plates were then subjected to 3 washes with PBST before blocking with 3% BSA in PBST at 37°C for 2 hours. Patient serum samples were diluted 1:1000 in PBST, added to the wells, and incubated for 3 hours at room temperature. Following 5 washes, HRP-conjugated anti-human IgG (1:4000 dilution; 709-036-098 Jackson ImmunoResearch Laboratories Inc.) was applied, and incubation proceeded for 1 hour. The reaction was developed with TMB substrate (Fisher Scientific Inc.), stopped with 1 M sulfuric acid, and absorbance was recorded at 450 nm.

### Immunohistochemical and Immunofluorescence staining.

IHC and immunofluorescence staining was performed on 4 μm–thick formalin-fixed, paraffin-embedded tissue sections. Sections were first deparaffinized in xylene for 10 min, washed with 100% ethanol followed by 95% and 70% ethanol, rinsed in distilled water and then washed in phosphate-buffered saline (PBS). For IHC staining, sections were incubated with H&E and Prussian blue (Sigma-Aldritch) following standard protocols. For immunofluorescence staining, samples were processed for antigen retrieval with DIVA Decloaker (DV2004, Biocare) using a digitally controlled pressure cooker as heat source, followed by a cooling-off period of 10 min. Blocking with 1% BSA (BP1600-100, Fisher Scientific) in PBS was performed for 1 hour at room temperature. For staining with recombinant mAb generated from intragraft PC, goat anti-human IgG (H+L) Fab fragment (109-007-003, Jackson ImmunoResearch Laboratories Inc.) was added to the blocking solution. Samples were washed in PBS for 5 min after the blocking step and primary antibody was added before incubation overnight at 4°C. After incubation, samples were washed thrice in PBS for 10 min, before secondary antibody was added and incubated for 1 hour at room temperature. Finally, DAPI staining and mounting of slides were performed with Invitrogen ProLong Gold Antifade DAPI mounting medium (P36941, Invitrogen). The following antibodies were used as primary antibodies in these experiments: anti-CD19 (NCL-L-CD19-163, Leica Biosystems), anti-CD138/Syndecan-1 (ab181789, Abcam), anti-CD3e (MA5-16622, Fisher Scientific), anti-CD68 (76437T, Fisher Scientific), anti-CD31 (ab199012, Abcam), anti-αSMA (14-9760-82, Fisher Scientific), anti-HO1 (MA1-112, Thermo Scientific), and anti-Bilirubin (CAU21553, Fisher Scientific) as well as the 28 recombinant mAb generated from intragraft PC through expression cloning (Genscript). Anti-human IgG DyLight 550 (SA5-10127, Invitrogen), anti-human IgG AF 647 (709-606-098, Jackson Labs), anti-mouse IgG AF 647 (4410S, Cell Signaling), anti-rabbit IgG (4412S, Cell Signaling), and anti-rat IgG AF 555 (4417S, Cell Signaling) were used as secondary antibodies. Images were taken using either a Leica DMI 6000B or a Zeiss LSM 900 microscope. To quantify the binding of the different mAb on CAV tissue, we measured the mean fluorescence intensity (MFI) using ImageJ (29). In brief, a raw MFI value was calculated for each mAb by analyzing an equal-sized staining image based on the specific mAb, in the same tissue area. This raw MFI was then normalized by subtracting the background signal created by the secondary antibody alone. For comparison, both MFI and ELISA data were z-transformed to ensure scale-independent comparability. To assess the correspondence between both methods, we calculated the spearman rank correlation coefficient, which measures the strength and direction of the relationship based on their rank orders, using GraphPad Prism (GraphPad Software, Inc., 2024) (*r* = 0.4336, *P* = 0.0211).

### Elution of IgG from cardiac tissue and assessment of reactivity to bilirubin.

Snap frozen tissues from 5 explanted CAV hearts and 5 non-CAV control hearts of similar weight were minced and homogenized in 500 μL of extraction buffer (100 mM Tris-HCl, pH 7.4, 150 mM NaCl, 1% Triton X-100, 1 mM EDTA, and 1X protease inhibitor cocktail). Homogenates were incubated on an orbital shaker for 2 hours at 4 °C, followed by centrifugation at 15,000*g* for 20 min. The resulting supernatants were collected and stored at −80 °C until further analysis. Total IgG concentration in tissue extracts was quantified using the LEGEND MAX Human Total IgG ELISA Kit (BioLegend, San Diego, CA, USA). Antibody reactivity to unconjugated bilirubin was assessed by ELISA using serial dilutions of extracted supernatants. Reactivity values were corrected by subtracting the DMSO background signal (the solvent used for unconjugated bilirubin) and normalized for IgG concentration in each extract. Comparisons between CAV and non-CAV groups were performed using the total peak area under the curve (AUC) for each sample, with statistical significance determined by Student’s *t* test.

### Statistics.

Statistical analyses were performed using R software v4.3.1 and GraphPad Prism 10. *P* values < 0.05 were considered statistically significant. Fisher’s exact tests were used to compare differences in cell type distributions and VH gene usage between CAV and PBMC. Gene set enrichment analyses were performed using the Fast Gene Set Enrichment Analysis package v1.28.0 for R using the KEGG, GOPB, Hallmark and Reactome collections from the Molecular Signature Database (MSigDB) v2023.2 (http://software.broadinstitute.org/gsea/msigdb/index.jsp). Benjamini-Hochberg correction was applied to adjust for multiple testing. ELISA data underwent standardization, unsupervised clustering, and classification using the Boruta feature selection algorithm (version 8.0.0) in R (version 4.3.1). Principal component analyses (PCAs) were generated using the Plotly package (version 4.10.4).

### Study approval.

The study was approved by the Columbia University Internal Review Board.

### Data availability.

The CAV scRNA-seq data were deposited into GEO with the accession number GSE293483. The study also utilized existing publicly available PBMC scRNA-seq data (GEO accession GSM4560070, SRA accession SRP073767 and https://support.10xgenomics.com/single-cell-gene-expression/datasets/3.0.0/pbmc_10k_v3). The R codes for scRNA-seq data analysis were uploaded to GitHub repository: https://github.com/sarahbsee/CAV_scRNAseq_analysis/blob/main/CAV_scRNAseq_analysis.R (commit ID 3ad7c83720effb670b43aef4660c4386f3bafbfb). Values for data points in graphs and tables are reported in the Supporting Data Values file.

## Author contributions

SBS, TA, SM, KJC, JCM, RGK, OB, EPK, and EZ wrote the manuscript. SBS, TA, MD, MA, HTTN, SM, LD, HC, PR, LR, RGK, and OB conducted the experiments. SBS, TA, MD, MA, HTTN, SM, LD, HC, PR, LR, RV, and KJC acquired the data. SBS, TA, MD, MA, HTTN, SM, HC, LD, LR, CCM, MJS, RV, KJC, RGK, and EZ analyzed the data. CCM, MJS, AO, JGC, MF, EPK, YN, KT, RV, and OB obtained or provided samples and/or reagents. SBS, TA, SM, KJC, GB, JCM, RGK, OB, EPK, and EZ designed research studies.

## Funding support

This work is the result of NIH funding in part, and is subject to the NIH Public Access Policy. Through acceptance of this federal funding, the NIH has been given a right to make the work publicly available in PubMed Central.

- Research grants AI116814, AI154845, AI184963 and AI176507 (to EZ).
- Research grant HL148528 (to KJC).
- Research grant RYC2022-036797-I (to HC).
- NIH/NCI Cancer Center Support Grant P30CA013696.
- The National Center for Advancing Translational Sciences, National Institutes of Health, Grant Number UL1TR001873.
- Office of the Director, National Institutes of Health award S10OD020056.

## Supplementary Material

Supplemental data

ICMJE disclosure forms

Supporting data values

## Figures and Tables

**Table 2:**
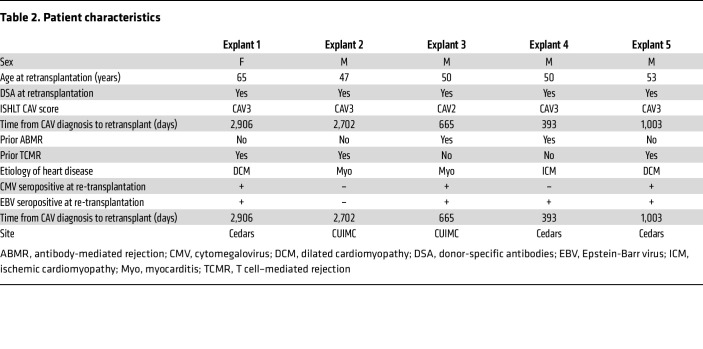
Patient characteristics

**Table 1:**
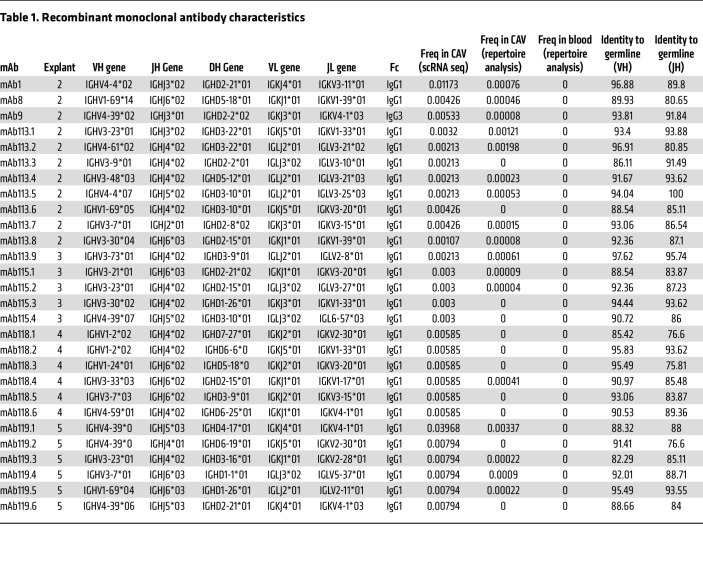
Recombinant monoclonal antibody characteristics

## Version 1 11/25/2025

In-Press Preview

## Version 2 02/02/2026

Electronic publication
