# Supplementary material for: Dominant intragraft plasma cells targeting bilirubin implicate local heme catabolism in human cardiac allograft vasculopathy
Source: J Clin Invest. 2025 Nov 25;136(3):e194138. doi: 10.1172/JCI194138 (PMC12867138; doi:10.1172/JCI194138)
Supplement: Supplemental data [file jci-136-194138-s148.pdf]

Supplementary Materials

Table S1. Recombinant monoclonal antibody characteristics.

Table S2. Analysis of clonally-related V-J IGHV sequences in CAV B cells, CAV PC and PBMC.

Table S3. Chemical adduct panel.

Table S4. Summary of bilirubin immunofluorescence staining.

Table S5. Longitudinal assessment of endomyocardial biopsies collected from patient 2.

Table S6. Clinical annotations of heart transplant recipients with or without CAV included in the cross-sectional assessment of serum anti-bilirubin IgG levels.

Figure S1. Pseudotime trajectory analysis of B cells and PC from combined CAV and PBMC, CAV only and PBMC only. The root nodes were selected based on expression the naïve B cell markers IGHD, SELL and CCR7.

Figure S2. Expression profile of 10 PC markers in Pb/PC populations from CAV and healthy donor PBMC.

Figure S3. Top 20 Hallmark and KEGG molecular signatures of total B cells and plasmablast/PC in the graft compared to healthy PBMC.

Figure S4. A) UMAP embeddings of Pb/PC subclusters (0 and 1) from combined CAV and PBMC, CAV only and PBMC only. B) Heat map showing the top differentially expressed genes from Pb/PC subcluster 0 compared to subcluster 1. Genes associated with cell cycle/proliferation,

B-cell receptor signaling and plasmablast/early plasma cells were upregulated in cluster 0. Genes associated with unfolded protein response; protein secretion and late plasma cells were upregulated in cluster 1.

Figure S5. Reactivity of mAb generated from intragraft PC to HLA assessed by complement-dependent cytotoxicity. Control HLA-class I-reactive mAB W6/32 was included

Figure S6. Reactivity of 96 recombinant mAb generated from CD19+CD27+ memory B cells from the blood of patients 2 and 3 to bilirubin assessed by ELISA. Bilirubin-reactive (mAb9) and non-reactive (mAb1) mAb were used as positive and negative controls respectively.

Figure S7. Prussian blue, Hematoxylin & Eosin staining and immunofluorescence staining for bilirubin of FFPE sections from 5 cardiac grafts without CAV.

Figure S8. Immunofluorescence staining for CD19, CD3, CD138 and bilirubin of FFPE sections from 4 endomyocardial biospies with evidence of immune infiltrates collected from patient 2 18-4 months before re-transplantation.

Figure S9. Immunofluorescence staining of FFPE sections from a representative explanted cardiac graft with CAV with 28 recombinant human mAb generated from intragraft PC.

Figure S10. Prussian blue, Hematoxylin & Eosin staining of FFPE sections from 4 explanted cardiac grafts with CAV.

Figure S11. Bilirubinemia in CAV and non-CAV patients from the Columbia University Irving Medical Center and Vall d'Hebron University Hospital cohorts.

Figure S12. Reactivity of IgG eluted for cardiac explant with CAV (N=5) and control healthy cardiac tissue without CAV (N=5) to bilirubin assessed by ELISA.

Table S1. Recombinant monoclonal antibody characteristics

mAb	H-CDR3 nt	H-CDR3 aa
CAV124E11	TGTGCGAAAGACCCTGGCCCCGGTAACTGGAAGGAACAACCCTGG	CAKDPGPGNWKEQPW
CAV124F12	TGTGCGAGAGAGAACAAGGACCAACCAAATTGTGGTGGAATCTGCCATCCCCTGGCTTCCTGG	CARENKDQPNCGGICHPLASW
CAV124J4	TGTGCGACCGACCATTACTATGATAGTAGTGGTTATCATGGCGCTTCATTTTCCTGG	CATDHYYDSSGYHGASFSW
CAV124J4	TGTGCGACCGACCATTACTATGATAGTAGTGGTTATCATGGCGCTTCATTTTCCTGG	CATDHYYDSSGYHGASFSW
CAV124K5	TGTGGGAGAGACACTGGCATTTACGGGGGGGACTGGCTCGACCCCTGG	CGRDTGIYGGDWLDPW
CAV124K6	TGTGCAAAAGGGGAATGGCTGGGCACCTATGGCATGGACGTCTGG	CAKGEWLGTYGMDVW
CAV124L3	TGTGCTGGGGGGACGAAGTGGCTGGCTATCCCTTACTGG	CAGGTKWLAIPYW
CAV124L9	TGTGCGAAAGCCCTCAACACTGCGGGGAGTTTTTATCACCCGGAGGACCACTGG	CAKALNTAGSFYHPEDHW
CAV124M13	TGTGCGAGAAGCCGTGGTGGTAGCAGCAGCTGGCATGACTACTGG	CARSRGGSSSWHDYW
CAV124P5	TGTGCGAGATACTTAATCTACTACGGTATGGACGTCTGG	CARYLIYYGMDVW

Table S1. Recombinant monoclonal antibody characteristics (cont.)

mAb	H-CDR3 nt	L-CDR3 nt	H-CDR3 aa	L-CDR3 aa
mAb1	TGTGCGAGAGATTCTGGGGTTGCGGGTCTACGCGTCGGCGGCGGCTTTGATATCTGG	TGTCAGCAGCGTAGCAGCTGGCTTCTCACTTTC	CARDSGVAGLRVGGGFDIW	CQQRSSWLLTF
mAb8	TGTGCGAGAGCGGAGCGTGGATACAGCGTGGCTATCACCTATAAGAGCCTGGACTTTTACCTCGCCATGGACGTCTGG	TGTCAACAGAGTTACAGTAATACGTGGACGTTC	CARAERGYSVAITYKSLDFYLAMDVW	CQQSYSNTWTF
mAb9	TGTGCGAGAGCTAAGATTCAATATTGCAGTAGAACCAAGTGTTATAATGATGCTTTTGATCTCTGG	TGTCAGCAATATCATACAACCCCATTCACTTTC	CARAKIQYCSRTKCYNDAFDLW	CQQYHTTPFTF
mAb113.1	TGTGCGAAAAGCAAGACGTTTTACTATCATAGTAGTGGTTATTACTACGGTGCTTTTGATATCTGG	TGTCAACAGTATGCTAGTCTCCCCCTCACCTTC	CAKSKTFYYHSSGYYYGAFDIW	CQQYASLPLTF
mAb113.2	TGTGCGAGAGTTCCGTACGTTAGTATGATAGTATACTGG	TGTCAGGTGTGGGATAGTAATGGTGATCAAGTGGTATTC	CARVPYVSMIVYW	CQVWDSNGDQVVF
mAb113.3	TGTGCAAAGGGTTCTCTCCGAGTTCCCGCTGCCGCCCACGAGGACTACTTTGACCACTGG	TGTTACTCAATAGACAGCAGAACTAATGATAGAGTGTTC	CAKGSLRVPAAAHEDYFDHW	CYSIDSRTNDRVF
mAb113.4	TGTGCGAGCGAGTGGCTACGCAGCAATTTTGACTACTGG	TGTCAGGTGTGGGATAGTACCAATAATCATGTGCTCTTC	CASEWLRSNFDYW	CQVWDSTNNHVLF
mAb113.5	TGTGCGAGAGATGCCCCTGGTCCGTACAACTGGTTCGACCCCTGG	TGTCAATCAGCAGACAGCAATGGTATTGTGATTTTC	CARDAPGPYNWFDPW	CQSADSNGIVIF
mAb113.6	TGTGCGACTTCGGGGAGTTATTTTAACGAGAAGGAACCGTTTCACTTCTGG	TGTCAGCAGTATGGTGGCTCACCTCCTGTCACCTTC	CATSGSYFNEKEPFHFW	CQQYGGSPPVTF
mAb113.7	TGCGCGAGAGGAGGTGGCTTATTTTGTACTGGTCGTGTTTGCCTGCAGTACACATACTTCGATCTCTGG	TGTCTGCAATATAGTATCTGGCCTACCTTC	CARGGGLFCTGRVCLQYTYFDLW	CLQYSIWPTF
mAb113.8	TGTGCGAGAGATGGCAACAAATGTAGTGGTGGTAGGTGCCAGTATTACTTCCACTACATGGACGTCTGG	TGTCAACAGAGTTACAATACCCCGGTGTTC	CARDGNKCSGGRCQYYFHYMDVW	CQQSYNTPVF
mAb113.9	TGTACTAGAATGGGACCTCGATACACTGGCTGGTACTTTGACTCCTGG	TGCAGCTCATATGCAGGCAGCAACTATGTGATATTC	CTRMGPRYTGWYFDYW	CSSYAGSNYVIF
mAb115.1	TGCGCGAGAGACTATTGCAGTGGTGGTGACTGCCTCTCCGGTTTCTTCTATTACTACATGGACCTCTGG	TGTCAGTACTATGGGAGTTCACGCACGTGGACGTTC	CARDYCSGGDCLSGFFYYYMDLW	CQYYGSSRTWTF
mAb115.2	TGTGCGTCCCGGCGGGAGGAGTTTGACTGCTGG	TGTTATTCTGCGGCTGACACCTATCGGGTGTTC	CASRREEFDCW	CYSAADTYRVF
mAb115.3	TGTGCGAAGCATCAGCGGCCTGGGAGCTACGACAAACCCTTTGACTACTGG	TGTCAACAGTATGATAATTTATTCACTTTC	CAKHQRPGSYDKPFDYW	CQQYDNLFTF
mAb115.4	TGTGCGAGAAGCTTTCATCAGTTCCAACTCTGGTATGGGGGCCGGTTCGACCCCTGG	TGTCAGTCTTATGATAGCAGCAATCCTTGGGTGTTC	CARSFHQFQLWYGGRFDPW	CQSYDSSNPWVF
mAb118.1	TGCACGAGAGCATACTTGGGAATTGGGGGTTACTGG	TGTATGCAAGGAACGTACTGGCCGTACACTTTT	CTRAYLGIGGYW	CMQGTYWPYTF
mAb118.2	TGTGCGAGAGAGCCCGGCCCCTTTGACTACTGG	TGTCAACAGTATGATGATCTCCCAAGGACCTTC	CAREPGPFDYW	CQQYDDLPRTF
mAb118.3	TGTGCAACAGAGGACACAGTTATGGTCCCTTACGGTCTGGACGTCTGG	TGTCAGCAGTATAATACCTCACCGTACACTTTT	CATEDTVMVPYGLDVW	CQQYNTSPYTF
mAb118.4	TGTGCGAGGGATGAGACGGTAGTGGTGCCCCCTGAGAGGGACCCTTACAATTTTGTGTCCAATTTTCAAAAGGGAGTCCACCACTTTTACGGTATGGACGTCTGG	TGTCTACAGCATAATAGTTACTTCACGTGGACGTTC	CARDETVVVPPERDPYNFVSNFQKGVHHFYGMDVW	CLQHNSYFTWTF
mAb118.5	TGTGCGAGAGGGCTTAGAGGAGAAAAATTACGATATTTTGACTGGCCGAGTTACTACGGGATGGACGTCTGG	TGTCAGCAGTATAATAACTGGCCTCCGCACACTTTT	CARGLRGEKLRYFDWPSYYGMDVW	CQQYNNWPPHTF

Table S1. Recombinant monoclonal antibody characteristics (cont.)

mAb	H-CDR3 nt	L-CDR3 nt	H-CDR3 aa	L-CDR3 aa
mAb118.6	TGTGCGGGGGACGGGGTGTACGGGCGCTTTGACTACTGG	TGTCAGCAATATTATACTACTCCGTGGACGTTC	CAGDGVYGRFDYW	CQQYYTTPWTF
mAb119.1	TGTGTCAGAGTGACCCTCGGTGACCACACACCCAACTATTTCGACTCCTGG	TGTCAACAATATTATATTACCCCTCTCACTTTC	CVRVTLGDHTPNYFDSW	CQQYYITPLTF
mAb119.2	TGTCCAGTGGCGGACCTCTACTGG	TGCATGCAAGCTACACACTGGCCTATCACCTTC	CPVADLYW	CMQATHWPITF
mAb119.3	TGTGCGAAGGGCTTTCTGACTTTGGGGGAAGTAGGTCCGTTTGACCAGTGG	TGCATGCAAGCTCTAGAAACTCCTCCGACGTTC	CAKGFLTLGEVGPFDQW	CMQALETPPTF
mAb119.4	TGTGCGAGAGGACCATCAACTGGAACACTGGCCTATTACAACTACTACATGGACGTCTGG	TGTATGGTTTGGCCAAGCAATGCTTTGGGGGTGTTC	CARGPSTGTLAYYNYYMDVW	CMVWPSNALGVF
mAb119.5	TGTGCGAGCGAGGGGGAGGCTTTGAGTGTGAGCTACGGAAACGACTACTACTACTACATGGACGTCTGG	TGCTGCTCATATGCAGGCACTTACACTTTGATATTC	CASEGEALSVSYGNDYYYYMDVW	CCSYAGTYTLIF
mAb119.6	TGTACGAGAGCAGTGATTGCTGACCATACCCCCGGTTGGTTCGACACCTGG	TGTCAGCAATATTATTTTACTCCGCTCACTTTC	CTRAVIADHTPGWFDTW	CQQYYFTPLTF

Table S2. Clonally-related IGHV rearrangements in CAV and PBMC

**Cell populations**	**Number of sequences with shared V-J usage**	**Number of clonally related sequences**
CAV PC vs PBMC	239	1
CAV B cell vs PBMC	1384	6
CAV PC vs CAV B cell	164	62

Table S3: Adducts and antigens used in ELISA.

Adducts	Amino acid modify	Peptide Sequence	Formula	M.W.	Purity	Source	Coating concentration
**Unmodified Lysine**	K	H2N-RRKRR-OH	C30H62N18O6	770.93	0.9931	Abclonal Science	10 μM
Monomethyl-Lys	K	H2N-RRK(Methylation)RR-OH	C31H64N18O6	784.92	0.9054	Abclonal Science	10 μM
Dimethyl-Lys	K	H2N-RRK(Dimethylation)RR-OH	C32H66N18O6	798.92	0.9297	Abclonal Science	10 μM
Trimethyl-Lys	K	H2N-RRK(Trimethylation)RR-OH	C33H68N18O6	812.92	0.9961	Abclonal Science	10 μM
Formyl-Lys	K	H2N-RRK(Formylation)RR-OH	C31H62N18O7	798.96	0.9181	Abclonal Science	10 μM
Propionyl-Lys	K	H2N-RRK(Propionylation)RR-OH	C33H66N18O7	826.93	0.9474	Abclonal Science	10 μM
Butyryl-Lys	K	H2N-RRK(Butyrylation)RR-OH	C34H68N18O7	841.04	0.9388	Abclonal Science	10 μM
2-Hydroxyisobutyryl-Lys	K	H2N-RRK(2-Hydroxyisobutyrylation)RR-OH	C34H68N18O8	857.03	0.9577	Abclonal Science	10 μM
Crotonyl-Lys	K	H2N-RRK(Crotonylation)RR-OH	C34H66N18O7	839.02	0.9873	Abclonal Science	10 μM
Malonyl-Lys	K	H2N-RRK(Malonylation)RR-OH	C33H64N18O9	856.99	0.9028	Abclonal Science	10 μM
Succinyl-Lys	K	H2N-RRK(Succinylation)RR-OH	C34H66N18O9	871.02	0.9808	Abclonal Science	10 μM
Glutaryl-Lys	K	H2N-RRK(Glutarylation)RR-OH	C35H68N18O9	885.04	0.9639	Abclonal Science	10 μM
Beta-hydroxybutyryl-Lys	K	H2N-RRK(β-hydroxybutyrylation)RR-OH	C34H68N18O8	857.02	0.9565	Abclonal Science	10 μM
Biotinyl-Lys	K	H2N-RRK(Biotilation)RR-OH	C40H76N20O8S1	997.19	0.9794	Abclonal Science	10 μM
Carbamyl-Lys	K	H2N-RR(Homocitrulline)RR-OH	C34H68N18O8	813.92	0.9358	Abclonal Science	10 μM
N-homocysteinyl-Lys	K	H2N-RRK(Homocystine)RR-OH	C34H69N19O7S1	888.07	0.9233	Abclonal Science	10 μM
Acetyl-Lys	K	H2N-K(Acetylation)x5-OH	n/a	869	>90%	New England pep	10 μM
MDA-Lys (Malondialdehyde)	K	H2N-K(MDALys)x5-OH	n/a	n/a	n/a	New England pep	10 μM
Pyrraline	K		C12H18N2O4	254.28	n/a	Toronto Research Chemicals	1 mM
CEL (N ε -(1-Carboxyethyl)-L-lysine)	K		C9H18N2O4	218.25	≥95%	Cayman Chemicals	1 mM
CML (Carboxymethyl-lysine)	K		C8H16N2O4	204.226	≥95%	Cayman Chemicals	1 mM
Fructose lysine	K		C12H24N2O7	308.331	0.9	Synthose	1 mM
NEDD8	K		n/a	8600	0.95	Fisher Scientific	1 μM
SUMO2	K		n/a	10600	n/a	Creative BioMart	1 μM
Protoporphyrin IX	K		C34H34N4O4	562.7	≥95%	Enzo Life Sciences	1 mM
Billirubin	K		C33H36N4O4	584.7	≥95%	Cayman Chemicals	1 mM
PLP (Pyridoxal phosphate - active form of vitamin B6)	K		C8H10NO6	265.2	≥95%	Cayman Chemicals	1 mM
**Unmodified Arginine**	R	H2N-RRRRR-OH	C30H62N20O6	798.94	0.9957	Abclonal Science	10 μM
Monomethyl-Arg	R	H2N-RRR(Methylation)RR-OH	C31H64N20O6	812.93	0.9711	Abclonal Science	10 μM
Asymmetric dimethyl-Arg	R	H2N-RRR(Asymmetric dimethylation)RR-OH	C30H62N22O6	826.93	0.9851	Abclonal Science	10 μM
Symmetric dimethyl-Arg	R	H2N-RRR(Symmetric Dimethylation)RR-OH	C32H66N20O6	826.93	0.9432	Abclonal Science	10 μM
Citrulline-Arg	R	H2N-RR(Cit)RR-OH	C30H61N19O7	799.94	0.9878	Abclonal Science	10 μM
MDA-Arg (Malondialdehyde)	R	H2N-K(MDAArg)x5-OH	n/a		n/a	Abclonal Science	10 μM
**Unmodified Serine**	S	H2N-RRSRR-OH	C27H55N17O7	729.83	0.9712	Abclonal Science	10 μM
Phosphoryl-Ser	S	H2NRRS(Phosphorylation)RR-OH	C27H56N17P1O10	809.83	0.9884	Abclonal Science	10 μM
GlcNAcyl-Ser (O-linked-N-acetylglucosaminylation)	S	H2N-RRS(GlcNAc)RR-OH	C35H68N18O12	933.14	0.9316	Abclonal Science	10 μM
GalNAcyl-Ser	S	H2N-RRS(GalNAc)RR-OH	C35H68N18O12	933.14	0.9012	Abclonal Science	10 μM
Pyruvoyl-Ser	S	Pyruvoyl-RRRR-OH (N-terminal Pyruvoyl)	C21H52N16O7	712.81	0.9159	Abclonal Science	10 μM
Phosphatidylserine	S		C42H78NO10P	788	≥97%	Millipore Sigma	1 mM
**Unmodified threonine**	T	H2N-RRTRR-OH	C28H57N17O7	743.86	0.9723	Abclonal Science	10 μM
Phosphoryl-Thr	T	H2N-RRT(Phosphorylation)RR-OH	C28H58N17O10P1	823.86	0.9708	Abclonal Science	10 μM
**Unmodified Histidine**	H	H2N-RRHRR-OH	C30H57N19O6	779.9	0.9595	Abclonal Science	10 μM
Methyl-His	H	H2N-RR(N-3-methyl histidine)RR-OH	C31H61N19O7	793.91	0.9822	Abclonal Science	10 μM
**Unmodified Methionine**	M	H2N-RRMRR-OH	C29H59N17S1O6	773.95	0.9995	Abclonal Science	10 μM
Sulfenyl-Met (SO)	M	H2N-RR(Methionine-sulfoxide)RR-OH	C29H59N17O7S1	789.95	0.9535	Abclonal Science	10 μM
Sulfinyl-Met (SO2)	M	H2N-RR(Methionine-sulfone)RR-OH	C29H59N17O8S1	805.95	0.9195	Abclonal Science	10 μM
Demethyl-Met (Homocysteine)	M	H2N-RR(Homocysteine)RR-OH	C28H57N17S1O6	759.9	0.9562	Abclonal Science	10 μM
**Unmodified Tyrosine**	Y	H2N-RRYRR-OH	C33H59N17O7	805.93	0.9862	Abclonal Science	10 μM
Nitrate-Tyr	Y	H2N-RRY(Nitration)RR-OH	C34H60N17O9	850.93	0.9661	Abclonal Science	10 μM
Chlorine-Tyr	Y	H2N-RR(3-chloro-tyrosine)RR-OH	C33H58N17O7C1	840.43	0.9687	Abclonal Science	10 μM
Hydroxyl-Tyr (L-DOPA)	Y	H2N-RR(L-DOPA; 3,4-dihydroxy)RR-OH	C33H59N11O18	821.93	0.9914	Abclonal Science	10 μM
Phosphoryl-Tyr	Y	H2NRRY(Phosphorylation)RR-OH	C33H60N17P1O10	885.93	0.9819	Abclonal Science	10 μM
Iodine-Tyr	Y	H2N-RR(3-Iodo-L-tyrosine)RR-OH	C33H58I1N17O7	931.79	0.9927	Abclonal Science	10 μM
Sulfonyl-Tyr (SO3)	Y	H2N-RRY(O-Sulfonylation)RR-OH	C33H59N17S1O10	885.93	0.972	Abclonal Science	10 μM
FMN (Flavin mononucleotide)	Y		C17H20N4O9P	514.4	≥95%	Cayman Chemical	1 mM
FAD (Flavin adenine dinucleotide)	Y		C27H31N9Na2O15P2	829.51	≥95%	Millipore Sigma	1 mM
**Unmodified Cystine**	C	H2N-RR(Cystine)RR-OH	C30H60N18S2O8	865.05	0.9801	Abclonal Science	10 μM
Cysteine	C	H2N-RRCRR-OH	C27H55N17S1O6	745.9	0.9793	Abclonal Science	10 μM
S-Homocysteinyl-Cys	C	H2N-RRC(Homocystine)RR-OH	C28H56N17O6S1	759.91	0.9817	Abclonal Science	10 μM
Glutathione-Cys	C	H2N-RRC(GSH)RR-OH	C36H68N20O12S2	1037.19	0.9485	Abclonal Science	10 μM
S-palmitoyl-Cys	C	H2N-RRC(palmitoylation)RR-OH	C43H73N17O7S1	984.42	0.9174	Abclonal Science	10 μM
Sulfonyl-Cys (SO3H)	C	H2N-RRC(Sulfonylation,SO3)RR-OH	C27H55N17O9S1	793.9	0.9373	Abclonal Science	10 μM
Succinyl-Cys	C	H2N-RR(S-2-succinyl cysteine)RR-OH	C31H59N17O9S1	846.08	0.9355	Abclonal Science	10 μM
**Unmodified Glycine**	G	H2N-GRRRR-OH	C26H53N17O6	699.81	0.9053	Abclonal Science	10 μM
N-myristoyl-Gly	G	(N-myristoylation)-GRRRR-OH	C40H79N17O7	910.16	0.9645	Abclonal Science	10 μM
**Unmodified Tryptophan**	W	H2N-RRWRR-OH	C35H60N18O6	828.97	0.9799	Abclonal Science	10 μM
Dioxygenyl-Trp	W	H2N-RR(Kynurenine)RR-OH	C48H70N18O9	833.21	0.9784	Abclonal Science	10 μM
**Unmodified Glutamic Acid**	E	H2N-RRERR-OH	C29H57N17O8	771.87	0.9915	Abclonal Science	10 μM
Gamma-carboxyl-Glu	E	H2N-RR(γGlu)RR-OH	C30H57N17O10	815.92	0.988	Abclonal Science	10 μM
**Other residues**							
Adenosine 5′-diphosphoribose (ADP-Ribose)			C15H23N5O14P2	559.32	≥93%	Millipore Sigma	1 mM
Ubiquitin			n/a	8.5 kDa	0.95	Fisher Scientific	1 μM
Small ubiquitin-related modifier 1 (SUMO1)			n/a	11.5 kDa	n/a	Creative BioMart	1 μM
Neu5Gc (N-Glycolylneuraminic acid)			C11H19NO10	382.4	n/a	GlycoNZ	1 mM
Neu5Ac (N-Acetylneuraminic acid)			C11H19NO4	471.5	n/a	GlycoNZ	1 mM
Ribose			C5H10O5	150.13	0.99	Millipore Sigma	1 mM
PQQ (Pyrroloquinoline quinone)			C16H6N2O8	330.2	≥95%	Cayman Chemical	1 mM
V B9 (Folic acid)			C19H23N7O6	445.43	≥65%	Millipore Sigma	1 mM
TPP (Thiamine pyrophosphate)			C12H19ClN4O7P2S	460.77	≥95%	Millipore Sigma	1 mM
V B12 (Cobalamin)			C63H88CoN14O14P	1355.4	≥95%	Cayman Chemical	1 mM
NAD (Nicotinamide adenine dinucleotide)			C21H27N7O14P2	663.4	0.9	Cayman Chemical	1 mM
ATP (Adenosine triphosphate)			C10H14N5O13P3	551.1	≥95%	Cayman Chemical	1 mM
CTP (Cytidine triphosphate)			C9H14N3O14P3	527.1	≥95%	Cayman Chemical	1 mM
SAM (S-Adenosyl methionine)			C15H23ClN6O5S	507.82	≥75%	Millipore Sigma	1 mM
V B1 (Thiamine)			C12H17N4OS	337.3	≥98%	Cayman Chemical	1 mM
V C (L-ascorbic acid)			C6H8O6	176.1	≥95%	Cayman Chemical	1 mM
Tetrahydrobiopterin			C9H15N5O3	314.17	n/a	Millipore Sigma	1 mM
VA Retinal			C20H28O	284.44	≥98%	Millipore Sigma	1 mM
V K2 (Menaquinone)			C31H40O2	444.7	≥98%	Cayman Chemical	1 mM
Co Q10 (Ubiquinone (coenzyme Q-10)			C59H90O4	863.34	≥98%	Millipore Sigma	1 mM
Co M (2-Sulfanylethanesulfonate (coenzyme M)			C2H5O3S2	164.2	≥95%	Cayman Chemical	1 mM
V B6 (Pyridoxine)			C8H11NO3	169.2	≥98%	Cayman Chemical	1 mM
4-HNE GSH (4-hydroxy Nonenal Glutathione)			C19H33N3O8S	577.6	≥95%	Cayman Chemical	1 mM
4-HNE cysteine (4-hydroxy Nonenal Mercapturic Acid)			C14H25NO5S	319.4	≥98%	Cayman Chemical	10 μM
M1G (pyrimido[1,2-a]purin-10(3H)-one)			$C_8H_5N_5O$	187.76	.	Toronto Research Chemicals	10 μM
Pentosidine			C17H26N6O4	378.433	≥98%	Cayman Chemical	10 μM
UDP-GlcNAc (UDP-N-acetyl-D-Glucosamine)			C17H25N3O17P2 • 2Na [XH2O]	651.3	≥95%	Cayman Chemical	10 μM
Co-Methyl (Methylcobalamin)			C63H91CoN13O14P	1344.4	.	Sigma	10 μM
Co-A (Coenzyme A)			C21H36N7O16P3S	767.535	≥90%	Cayman Chemical	10 μM
ADP (Adenosine diphosphate)			C10H15N5O10P2	427.2	≥95%	Sigma	10 μM
Ox GSH (Oxidized glutathione)			C20H32N6O12S2	612.63	≥98%	Sigma	10 μM
D-ribose 5-phosphate			C5H9Na2O8P · 2H2O	310.1	≥99%	Sigma	10 μM
AMP (Adenosine monophosphate)			C10H14N5O7P · H2O	365.24	≥97%	Sigma	10 μM
V K1 (Phytonadione)			C31H46O2	450.7	.	Sigma	10 μM
V K3 (Menadione)			C11H8O2	172.2	.	Sigma	10 μM

Table S4. Summary of bilirubin staining.

Explant	CAV ISHLT score	Graft infiltrate	Tunica media
1	3	+	+
2	3	+	-
3	2	+	+
4	3	+	-
5	3	+	-
6	3	+	-
7	3	+	-
8	3	+	+
9	3	+	+
10	3	+	-
11	3	+	+
12	3	+	+
13	3	+	-
14	3	+	+
15	2	+	+
16	3	+	-
Non-CAV control grafts			
1	N/A	-	-
2	N/A	-	-
3	N/A	-	-
4	N/A	-	-
5	N/A	-	-
Healthy heart autopsy specimen			
2	N/A	-	-
2	N/A	-	-
3	N/A	-	-
4	N/A	-	-
5	N/A	-	-

Table S5. Longitudinal assessment of endomyocardial biopsies collected from patient 2.

Biopsy	Time to re-transplantation	Immune infiltrates	CD3	CD19	CD138	Bilirubin	ISLHT grade
1	19y, 4mo, 13d	-					
2	18y, 11mo, 30d	-					
3	18y, 7mo, 12d	+	+	+	-	+	2R
4	18y, 0mo, 18d	-					
5	17y, 5mo, 22d	-					
6	15y, 10mo, 1d	-					
7	15y, 4mo, 14d	+	+	+	+	+	1R
8	12y, 9mo, 26d	-					
9	12y, 4mo, 9d	-					
10	10y, 10mo, 12d	-					
11	9y, 3mo, 22d	-					
12	8y, 2mo, 1d	+	+	-	+	+	0
13	4y, 4mo, 11d	+	+	+	+	+	1R
14	1y, 11mo, 20d	-					
15	1y, 4mo, 1d	-					
16	0y, 0mo, 8d	-					

Table S6. Serum IgG reactivity to bilirubin - patient characteristics

Subject ID	Sex	Age at sample collection	DSA at sample collection	ISHLT CAV score at sample collection	Bilirubinemia al sample collection mg/dL	Time from Tx at sample collection (months)	Prior ABMR	Prior TCMR	Etiology of heart disease
VDH16	M	66	No	0	0.9	160	No	Yes	Dilated Cardiomyopathy
VDH18	M	57	No	0	1.9	59	No	Yes	Ischemic Cardiomyopathy
VDH19	M	73	No	0	0.5	218	No	No	Hypertophic cardiomyopathy
VDH20	M	77	No	0	0.3	212	No	Yes	Ischemic Cardiomyopathy
VDH21	M	72	No	0	0.5	141	No	No	Valvular cardiomyopathy
VDH22	F	56	No	0	0.4	219	No	No	Chronic myocarditis
VDH23	M	63	No	0	0.7	201	No	Yes	Ischemic Cardiomyopathy
VDH24	M	86	No	0	0.6	237	No	No	Ischemic Cardiomyopathy
VDH25	F	61	No	0	0.5	105	No	No	Nonischemic Cardiomyopathy
VDH26	M	64	No	0	0.2	33	No	Yes	Ischemic Cardiomyopathy
VDH27	M	72	No	0	0.7	70	No	No	Ischemic Cardiomyopathy
VDH29	M	71	No	0	0.6	181	No	Yes	Ischemic Cardiomyopathy
VDH30	M	79	No	0	0.7	189	No	Yes	Nonischemic Cardiomyopathy
VDH31	M	47	No	0	0.6	215	No	Yes	Nonischemic Cardiomyopathy
VDH12	M	68	No	1	0.7	38	No	Yes	Ischemic Cardiomyopathy
VDH14	F	69	No	1	0.4	101	No	Yes	Dilated Cardiomyopathy
VDH17	M	74	No	1	0.5	204	No	Yes	Nonischemic Cardiomyopathy
VDH28	M	73	No	1	0.5	131	No	Yes	Ischemic Cardiomyopathy
VDH1	M	59	Yes	2	0.8	190	Yes	Yes	Ischemic Cardiomyopathy
VDH2	M	70	Yes	2	0.6	207	No	Yes	Valvular cardiomyopathy
VDH6	F	56	No	2	N/A	276	No	Yes	Hypertophic cardiomyopathy
VDH11	F	73	No	2	0.4	211	No	No	N/A
VDH3	M	71	No	3	0.7	151	No	Yes	Ischemic Cardiomyopathy
VDH4	M	59	Yes	3	0.2	206	No	Yes	Ischemic Cardiomyopathy
VDH5	M	70	No	3	0.8	213	No	Yes	Ischemic Cardiomyopathy
VDH7	M	73	No	3	0.9	211	No	No	Nonischemic Cardiomyopathy
VDH8	M	62	No	3	NA	224	No	No	Ischemic Cardiomyopathy
VDH9	M	65	No	3	0.6	206	No	No	Ischemic Cardiomyopathy
VDH10	M	67	No	3	0.4	150	No	Yes	Valvular cardiomyopathy
VDH13	F	76	No	3	0.7	215	No	No	Nonischemic Cardiomyopathy
VDH15	M	73	No	3	0.6	213	No	Yes	Nonischemic Cardiomyopathy
CU1	F	55	Yes	0	0.5	12	Yes	Yes	Giant Cell Myocarditis
CU2	F	36	Yes	0	0.7	11	No	Yes	familial cardiomyopathy
CU3	M	76	No	0	0.4	192	No	Yes	Ischemic Cardiomyopathy
CU4	M	49	No	0	0.5	0	No	No	Nonischemic Cardiomyopathy
CU5	M	27	No	0	0.5	37	No	No	Nonischemic Cardiomyopathy
CU6	M	68	No	0	0.7	49	No	No	Ischemic Cardiomyopathy
CU7	F	62	No	0	0.3	169	No	No	Giant Cell Myocarditis
CU8	M	46	No	0	0.9	148	No	Yes	Dilated Cardiomyopathy
CU9	M	69	No	0	0.3	107	No	No	Ischemic Cardiomyopathy
CU10	M	61	No	0	0.8	83	No	Yes	Dilated Cardiomyopathy
CU11	M	72	No	0	0.4	35	No	No	Nonischemic Cardiomyopathy
CU12	M	51	No	0	0.2	108	No	No	Sarcoid Cardiomyopathy
CU13	M	65	No	0	0.8	121	No	No	Nonischemic Cardiomyopathy
CU14	M	59	No	0	0.4	36	No	Yes	Dilated Cardiomyopathy
CU15	F	58	No	0	1.5	218	No	No	Dilated Cardiomyopathy
CU16	F	51	Yes	0	0.4	36	Yes	Yes	Congenital Heart Disease
CU17	M	58	Yes	0	0.5	96	No	Yes	Ischemic Cardiomyopathy
CU18	M	55	No	0	0.3	35	No	Yes	Dilated Cardiomyopathy
CU19	F	60	No	0	0.4	73	No	Yes	Dilated Cardiomyopathy
CU20	M	56	No	0	0.4	12	No	No	Nonischemic Cardiomyopathy
CU21	M	27	Yes	0	1.7	139	Yes	No	Nonischemic Cardiomyopathy
CU22	M	58	Yes	0	0.7	35	No	No	Nonischemic Cardiomyopathy
CU23	M	65	No	0	0.4	22	No	No	Transthyretin amyloidosis
CU24	F	66	No	0	0.5	205	No	No	Dilated Cardiomyopathy
CU25	M	50	Yes	0	0.7	361	No	No	Dilated Cardiomyopathy
CU26	M	37	Yes	0	2.2	81	No	Yes	Dilated Cardiomyopathy
CU27	M	76	No	3	0.5	144	No	Yes	Dilated Cardiomyopathy
CU28	M	35	Yes	3	0.4	103	No	No	Dilated Cardiomyopathy
CU29	M	65	No	3	0.6	281	No	Yes	Dilated Cardiomyopathy
CU30	M	42	No	3	0.7	262	No	Yes	Dilated Cardiomyopathy
CU31	M	30	Yes	2	0.5	142	No	Yes	Lymphocytic Cardiomyopathy
CU32	F	11	Yes	2	0.1	64	No	No	Cardiac transplant coronary disease
CU33	M	20	Yes	2	0.3	161	No	No	Giant Coronary Aneurysms,Kawaski Disease, Ischemic Cardiomyopathy
CU34	M	74	Yes	2	1.2	325	No	No	Dilated Cardiomyopathy
CU35	M	57	No	2	0.4	196	No	Yes	Nonischemic Cardiomyopathy
CU36	M	65	Yes	2	0.4	183	Yes	Yes	Ischemic Cardiomyopathy
CU37	M	27	Yes	2	0.6	253	No	No	Dilated Cardiomyopathy
CU38	M	70	Yes	2	0.6	244	No	No	Dilated Cardiomyopathy
CU39	M	64	No	2	0.5	171	No	No	Ischemic Cardiomyopathy
CU40	M	63	Yes	2	0.6	155	No	No	Dilated Cardiomyopathy
CU41	F	51	No	2	1.3	155	No	No	Congenital Heart Disease
CU42	F	26	No	2	0.4	309	No	No	Cardiomyopathy
CU43	F	68	Yes	2	1.2	72	No	Yes	Nonischemic Cardiomyopathy
CU44	F	19	No	2	0.1	215	No	Yes	Dilated Cardiomyopathy
CU45	F	58	No	2	2.5	327	No	No	Rheumatic Heart Disease
CU46	M	82	No	2	0.5	233	No	No	Ischemic Cardiomyopathy
CU47	F	69	No	2	0.5	214	No	Yes	Hypertophic cardiomyopathy
CU48	F	73	No	2	0.3	133	No	No	Nonischemic Cardiomyopathy
CU49	M	76	Yes	2	0.6	157	Yes	No	Ischemic Cardiomyopathy
CU50	F	73	No	2	0.3	166	No	No	Ischemic Cardiomyopathy
CU51	M	71	No	2	0.6	157	No	Yes	Ischemic Cardiomyopathy
CU52	M	50	Yes	2	0.2	148	No	Yes	Dilated Cardiomyopathy
CU53	M	63	Yes	3	0.6	155	No	No	Dilated Cardiomyopathy

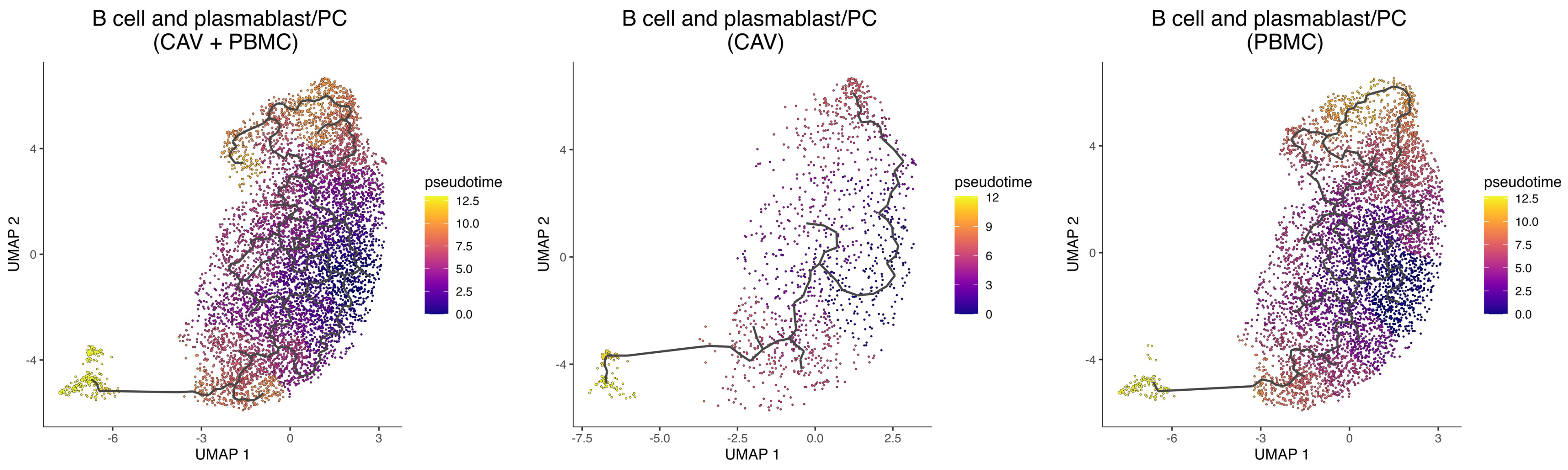

B cell and plasmablast/PC
(CAV + PBMC)
B cell and plasmablast/PC
(CAV)
B cell and plasmablast/PC
(PBMC)
UMAP 1
UMAP 2
pseudotime
12.5
10.0
7.5
5.0
2.5
0.0
12
9
6
3
0
-6
-3
0
3
4
-4
-7.5
-5.0
-2.5
0.0
2.5

Figure S1

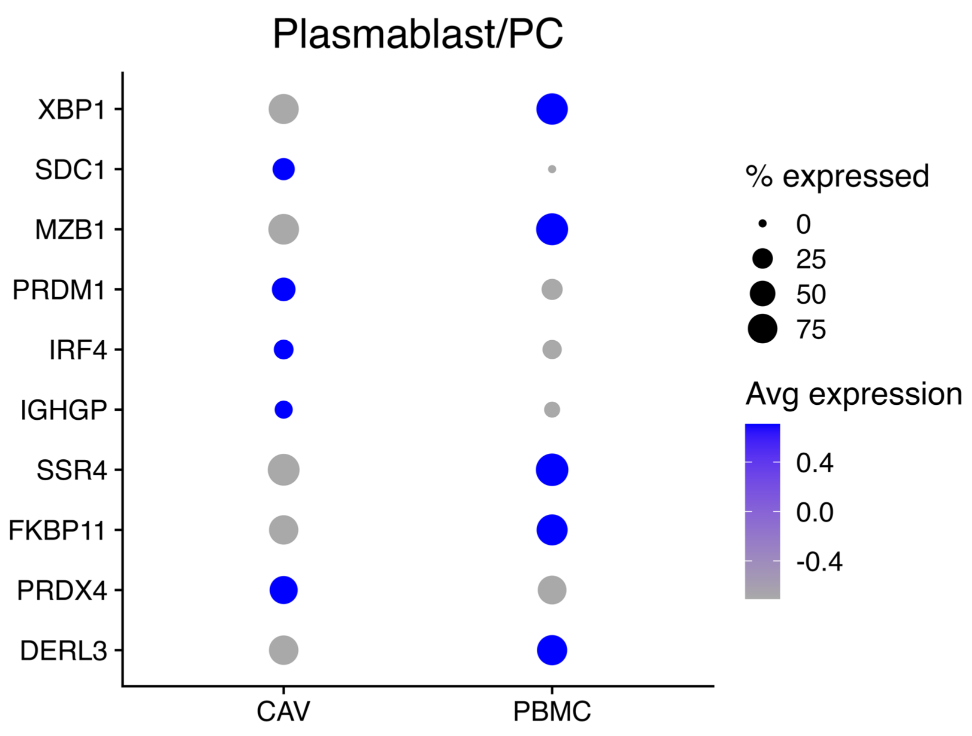
Plasmablast/PC
XBP1
SDC1
MZB1
PRDM1
IRF4
IGHGP
SSR4
FKBP11
PRDX4
DERL3
CAV
PBMC
% expressed
0
25
50
75
Avg expression
0.4
0.0
-0.4

Figure S2

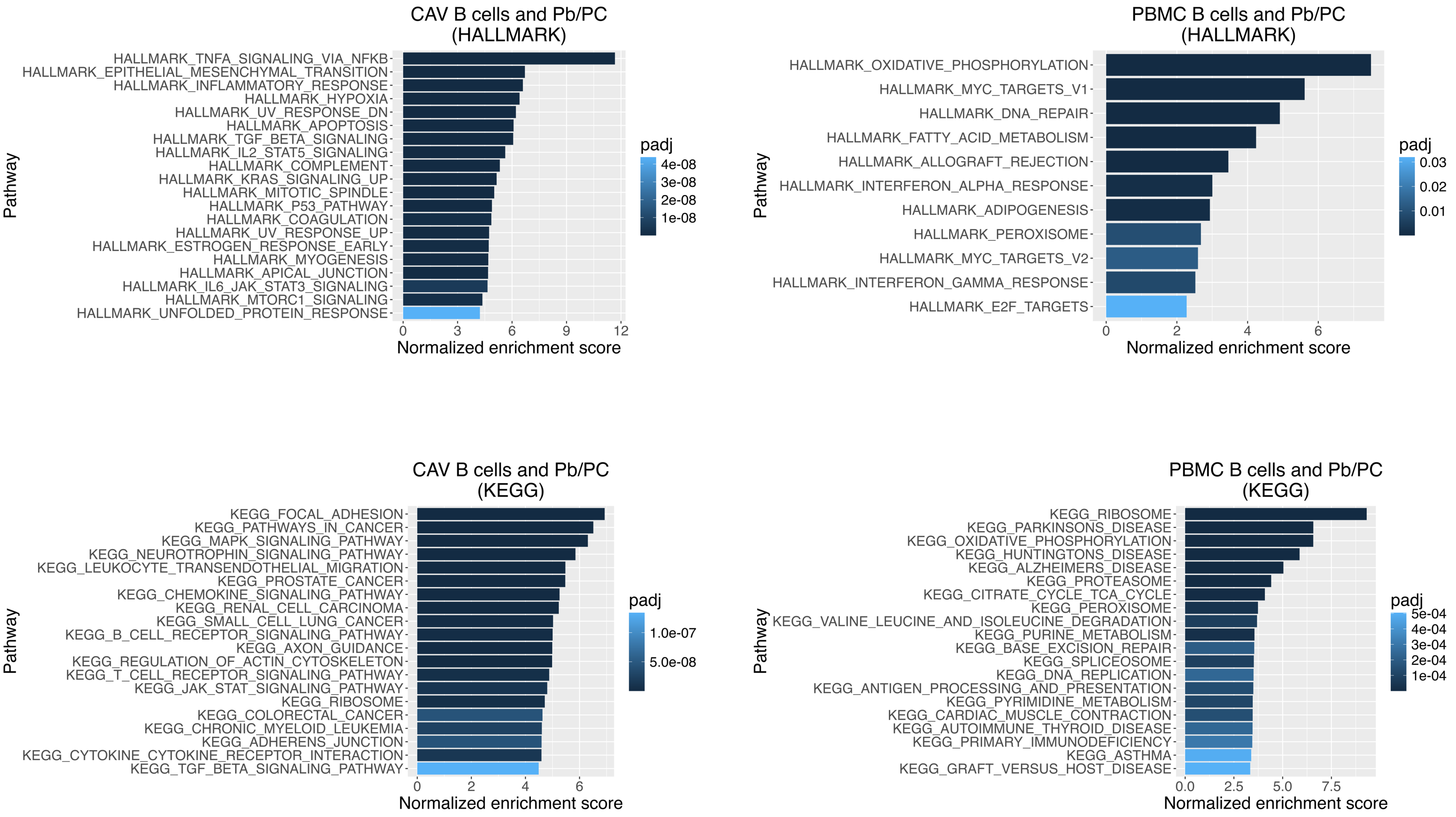
CAV B cells and Pb/PC (HALLMARK)
HALLMARK_TNFA_SIGNALING_VIA_NFKB
HALLMARK_EPITHELIAL_MESENCHYMAL_TRANSITION
HALLMARK_INFLAMMATORY_RESPONSE
HALLMARK_HYPOXIA
HALLMARK_UV_RESPONSE_DN
HALLMARK_APOPTOSIS
HALLMARK_TGF_BETA_SIGNALING
HALLMARK_IL2_STAT5_SIGNALING
HALLMARK_COMPLEMENT
HALLMARK_KRAS_SIGNALING_UP
HALLMARK_MITOTIC_SPINDLE
HALLMARK_P53_PATHWAY
HALLMARK_COAGULATION
HALLMARK_UV_RESPONSE_UP
HALLMARK_ESTROGEN_RESPONSE_EARLY
HALLMARK_MYOGENESIS
HALLMARK_APICAL_JUNCTION
HALLMARK_IL6_JAK_STAT3_SIGNALING
HALLMARK_MTORC1_SIGNALING
HALLMARK_UNFOLDED_PROTEIN_RESPONSE
Pathway
0
3
6
9
12
Normalized enrichment score
padj
4e-08
3e-08
2e-08
1e-08
PBMC B cells and Pb/PC (HALLMARK)
HALLMARK_OXIDATIVE_PHOSPHORYLATION
HALLMARK_MYC_TARGETS_V1
HALLMARK_DNA_REPAIR
HALLMARK_FATTY_ACID_METABOLISM
HALLMARK_ALLOGRAFT_REJECTION
HALLMARK_INTERFERON_ALPHA_RESPONSE
HALLMARK_ADIPOGENESIS
HALLMARK_PEROXISOME
HALLMARK_MYC_TARGETS_V2
HALLMARK_INTERFERON_GAMMA_RESPONSE
HALLMARK_E2F_TARGETS
Pathway
0
2
4
6
Normalized enrichment score
padj
0.03
0.02
0.01
CAV B cells and Pb/PC (KEGG)
KEGG_FOCAL_ADHESION
KEGG_PATHWAYS_IN_CANCER
KEGG_MAPK_SIGNALING_PATHWAY
KEGG_NEUROTROPHIN_SIGNALING_PATHWAY
KEGG_LEUKOCYTE_TRANSENDOTHELIAL_MIGRATION
KEGG_PROSTATE_CANCER
KEGG_CHEMOKINE_SIGNALING_PATHWAY
KEGG_RENAL_CELL_CARCINOMA
KEGG_SMALL_CELL_LUNG_CANCER
KEGG_B_CELL_RECEPTOR_SIGNALING_PATHWAY
KEGG_AXON_GUIDANCE
KEGG_REGULATION_OF_ACTIN_CYTOSKELETON
KEGG_T_CELL_RECEPTOR_SIGNALING_PATHWAY
KEGG_JAK_STAT_SIGNALING_PATHWAY
KEGG_RIBOSOME
KEGG_COLORECTAL_CANCER
KEGG_CHRONIC_MYELOID_LEUKEMIA
KEGG_ADHERENS_JUNCTION
KEGG_CYTOKINE_CYTOKINE_RECEPTOR_INTERACTION
KEGG_TGF_BETA_SIGNALING_PATHWAY
Pathway
0
2
4
6
Normalized enrichment score
padj
1.0e-07
5.0e-08
PBMC B cells and Pb/PC (KEGG)
KEGG_RIBOSOME
KEGG_PARKINSONS_DISEASE
KEGG_OXIDATIVE_PHOSPHORYLATION
KEGG_HUNTINGTONS_DISEASE
KEGG_ALZHEIMERS_DISEASE
KEGG_PROTEASOME
KEGG_CITRATE_CYCLE_TCA_CYCLE
KEGG_PEROXISOME
KEGG_VALINE_LEUCINE_AND_ISOLEUCINE_DEGRADATION
KEGG_PURINE_METABOLISM
KEGG_BASE_EXCISION_REPAIR
KEGG_SPLICEOSOME
KEGG_DNA_REPLICATION
KEGG_ANTIGEN_PROCESSING_AND_PRESENTATION
KEGG_PYRIMIDINE_METABOLISM
KEGG_CARDIAC_MUSCLE_CONTRACTION
KEGG_AUTOIMMUNE_THYROID_DISEASE
KEGG_PRIMARY_IMMUNODEFICIENCY
KEGG_ASTHMA
KEGG_GRAFT_VERSUS_HOST_DISEASE
Pathway
0.0
2.5
5.0
7.5
Normalized enrichment score
padj
5e-04
4e-04
3e-04
2e-04
1e-04

Figure S3

A

B

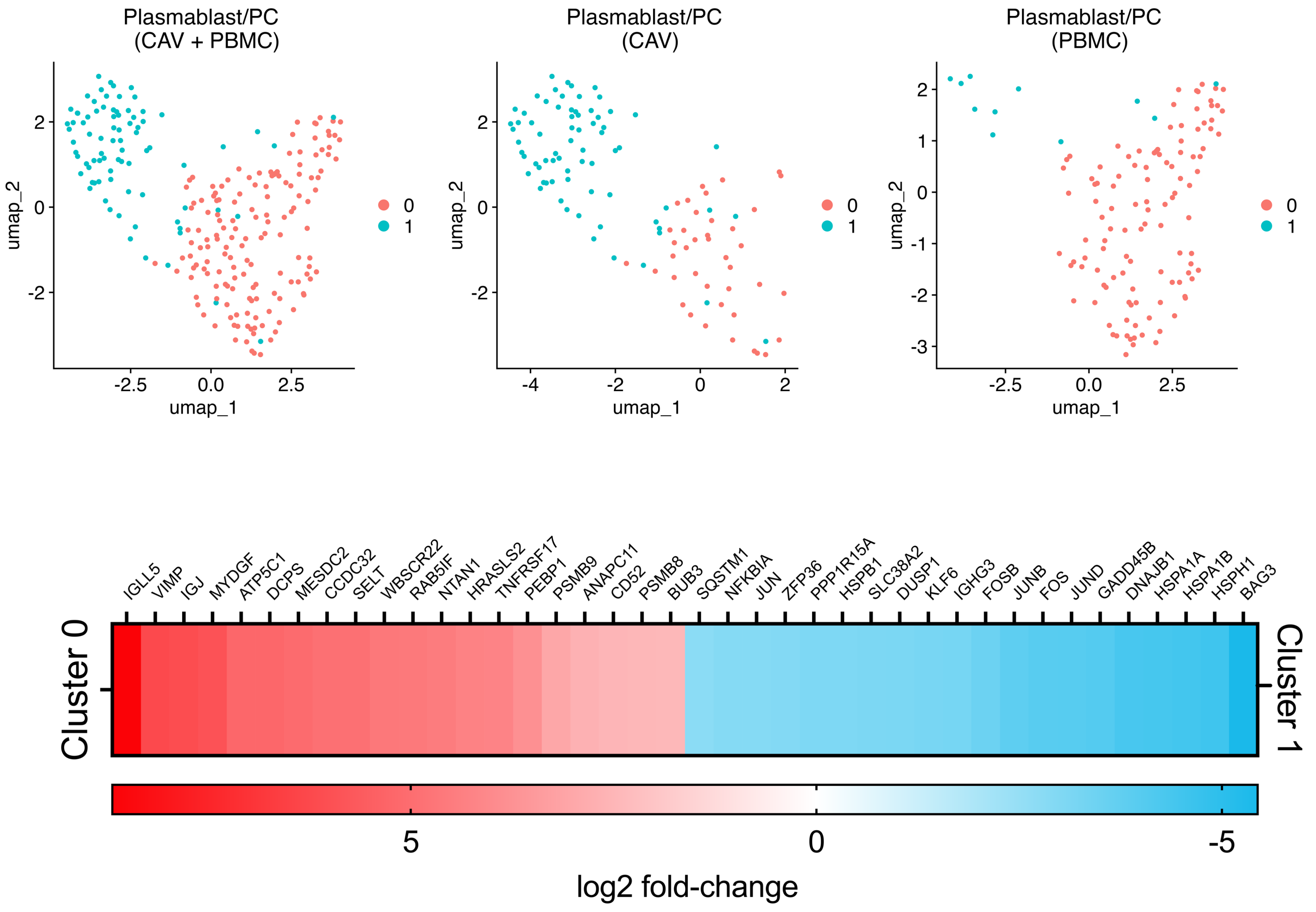

Up in Pb/PC cluster 0
Cell cycle/proliferation genes
B cell receptor signaling genes
Genes associated with Pb/early PC

Up in Pb/PC cluster 1
Unfolded protein response
Protein secretion
Genes associated with late PC

Figure S4

NewYork-Presbyterian

COLUMBIA

COLUMBIA UNIVERSITY IRVING MEDICAL CENTER

Immunogenetics and Cellular Immunology
Department of Pathology & Cell Biology
Columbia University Irving Medical Center
630 West 168th Street,
Vanderbilt Clinic, 15th Floor, VC 15-204
New York, NY 10032

Name: 113.1, 113.1
MRN: 113.1
DOB:
Category: Kidn
Physician: Mary
Status: Inact

Histocompatibility Laboratory Summary Report

Heart Pre-Transplant Evaluation

HLA Typing Results

Name/ID	A	B	Bw	Cw	DR	DRw	DQ	DQA1*	DPB1*	DPA1*	Tested Date
113.1, 113.1											
113.1											

DP Beta results are reported using EDP nomenclature.

PRA%: 0 **cPRA%:**

Histocompatibility Laboratory Summary Report

Heart Pre-Transplant Evaluation

HLA Typing Results

Name/ID	A	B	Bw	Cw	DR	DRw	DQ	DQA1*	DPB1*	DPA1*	Tested Date
113.2, 113.2											
113.2											

DP Beta results are reported using EDP nomenclature.

PRA%: 0 **cPRA%:**

Histocompatibility Laboratory Summary Report

HLA Typing Results

Name/ID	Sample Date / Sample Source / Receive Date / Tested Date	A*	B*	Bw	C*	DRB1*	DRB345*	DQB1*	DQA1*	DPB1*	DPA1*
E11, E11		()	()		()	()		()			
		()	()		()	()		()			

HLA typing was performed by NGS, PCR-SSOP, and/or qPCR to obtain the required resolution.
HLA serological equivalencies are displayed in parentheses.

PRA%: 0 cPRA%:

Histocompatibility Laboratory Summary Report

HLA Typing Results

Name/ID	Sample Date / Sample Source / Receive Date / Tested Date	A*	B*	Bw	C*	DRB1*	DRB345*	DQB1*	DQA1*	DPB1*	DPA1*
F12, F12		()	()		()	()		()			
		()	()		()	()		()			

HLA typing was performed by NGS, PCR-SSOP, and/or qPCR to obtain the required resolution.
HLA serological equivalencies are displayed in parentheses.

PRA%: 0 cPRA%:

Histocompatibility Laboratory Summary Report

HLA Typing Results

Name/ID	Sample Date / Sample Source / Receive Date / Tested Date	A*	B*	Bw	C*	DRB1*	DRB345*	DQB1*	DQA1*	DPB1*	DPA1*
J4, J4		()	()		()	()		()			
		()	()		()	()		()			

HLA typing was performed by NGS, PCR-SSOP, and/or qPCR to obtain the required resolution.
HLA serological equivalencies are displayed in parentheses.

PRA%: 0 cPRA%:

Histocompatibility Laboratory Summary Report

HLA Typing Results

Sample Date
Sample Source
Receive Date
Tested Date

Name/ID
K5, K5

A*	B*	Bw	C*	DRB1*	DRB345*	DQB1*	DQA1*	DPB1*	DPA1*
()	()		()	()		()			
()	()		()	()		()			

HLA typing was performed by NGS, PCR-SSOP, and/or qPCR to obtain the required resolution.
HLA serological equivalencies are displayed in parentheses.

PRA%: 0 cPRA%:

Histocompatibility Laboratory Summary Report

HLA Typing Results

Sample Date
Sample Source
Receive Date
Tested Date

Name/ID
K6, K6

A*	B*	Bw	C*	DRB1*	DRB345*	DQB1*	DQA1*	DPB1*	DPA1*
()	()		()	()		()			
()	()		()	()		()			

HLA typing was performed by NGS, PCR-SSOP, and/or qPCR to obtain the required resolution.
HLA serological equivalencies are displayed in parentheses.

PRA%: 0 cPRA%:

Histocompatibility Laboratory Summary Report

HLA Typing Results

Sample Date
Sample Source
Receive Date
Tested Date

Name/ID
L3, L3

A*	B*	Bw	C*	DRB1*	DRB345*	DQB1*	DQA1*	DPB1*	DPA1*
()	()		()	()		()			
()	()		()	()		()			

HLA typing was performed by NGS, PCR-SSOP, and/or qPCR to obtain the required resolution.
HLA serological equivalencies are displayed in parentheses.

PRA%: 0 cPRA%:

Histocompatibility Laboratory Summary Report

HLA Typing Results

Sample Date
Sample Source
Receive Date
Tested Date

Name/ID
L9, L9

A*	B*	Bw	C*	DRB1*	DRB345*	DQB1*	DQA1*	DPB1*	DPA1*
()	()		()	()		()			
()	()		()	()		()			

HLA typing was performed by NGS, PCR-SSOP, and/or qPCR to obtain the required resolution.
HLA serological equivalencies are displayed in parentheses.

PRA%: 0 cPRA%:

Histocompatibility Laboratory Summary Report

HLA Typing Results

Sample Date
Sample Source
Receive Date
Tested Date

Name/ID
M13, M13

A*	B*	Bw	C*	DRB1*	DRB345*	DQB1*	DQA1*	DPB1*	DPA1*
()	()		()	()		()			
()	()		()	()		()			

HLA typing was performed by NGS, PCR-SSOP, and/or qPCR to obtain the required resolution.
HLA serological equivalencies are displayed in parentheses.

PRA%: cPRA%:

New York, NY 10032

Histocompatibility Laboratory Summary Report

HLA Typing Results

Sample Date
Sample Source
Receive Date
Tested Date

Name/ID
P5, P5

A*	B*	Bw	C*	DRB1*	DRB345*	DQB1*	DQA1*	DPB1*	DPA1*
()	()		()	()		()			
()	()		()	()		()			

HLA typing was performed by NGS, PCR-SSOP, and/or qPCR to obtain the required resolution.
HLA serological equivalencies are displayed in parentheses.

PRA%: 0 cPRA%:

Histocompatibility Laboratory Summary Report

HLA Typing Results

Name/ID	Sample Date<br>Sample Source<br>Receive Date<br>Tested Date	A*	B*	Bw	C*	DRB1*	DRB345*	DQB1*	DQA1*	DPB1*	DPA1*
W6, W6		()<br>()	()<br>()		()<br>()	()<br>()		()<br>()			

HLA typing was performed by NGS, PCR-SSOP, and/or qPCR to obtain the required resolution.
HLA serological equivalencies are displayed in parentheses.

PRA%: 99 cPRA%:

	A	B	C	D	E	F	
1	1<br>NEGATIVE CONTROL<br>T B<br>-5 -5<br>-5 -5	2<br>POSITIVE CONTROL<br>99 99<br>99 99	3<br>Ia CONTROL<br>-5 99<br>-5 99	4<br>113.1<br>113.1<br>190000-00-01 KI<br>1/15/25<br>-5 -5<br>-5 #N/A -5	5<br>113.2<br>113.2<br>190000-00-02 KI<br>1/15/25<br>-5 -5<br>-5 #N/A -5	6<br>113.3<br>113.3<br>190000-00-03 KI<br>1/15/25<br>-5 -5<br>-5 #N/A -5	1
2	12<br>113.9<br>113.9<br>190000-00-09 KI<br>1/15/25<br>-5 -5<br>-5 #N/A -5	11<br>113.8<br>113.8<br>190000-00-08 KI<br>1/15/25<br>-5 -5<br>-5 #N/A -5	10<br>113.7<br>113.7<br>190000-00-07 KI<br>1/15/25<br>-5 -5<br>-5 #N/A -5	9<br>113.6<br>113.6<br>190000-00-06 KI<br>1/15/25<br>-5 -5<br>-5 #N/A -5	8<br>113.5<br>113.5<br>190000-00-05 KI<br>1/15/25<br>-5 -5<br>-5 #N/A -5	7<br>113.4<br>113.4<br>190000-00-04 KI<br>1/15/25<br>-5 -5<br>-5 #N/A -5	2
3	13<br>115.1<br>115.1<br>190000-00-10 KI<br>1/15/25<br>-5 -5<br>-5 #N/A -5	14<br>115.2<br>115.2<br>190000-00-11 KI<br>1/15/25<br>-5 -5<br>-5 #N/A -5	15<br>115.3<br>115.3<br>190000-00-12 KI<br>1/15/25<br>-5 -5<br>-5 #N/A -5	16<br>115.4<br>115.4<br>190000-00-13 KI<br>1/15/25<br>-5 -5<br>-5 #N/A -5	17<br>Quality Negative Control<br>-5 -5<br>-5 -5	18<br>118.1<br>118.1<br>190000-00-14 KI<br>1/15/25<br>-5 -5<br>-5 #N/A -5	3
4	24<br>119.1<br>119.1<br>190000-00-20 KI<br>1/15/25<br>-5 -5<br>-5 #N/A -5	23<br>118.6<br>118.6<br>190000-00-19 KI<br>1/15/25<br>-5 -5<br>-5 #N/A -5	22<br>118.5<br>118.5<br>190000-00-18 KI<br>1/15/25<br>-5 -5<br>-5 #N/A -5	21<br>118.4<br>118.4<br>190000-00-17 KI<br>1/15/25<br>-5 -5<br>-5 #N/A -5	20<br>118.3<br>118.3<br>190000-00-16 KI<br>1/15/25<br>-5 -5<br>-5 #N/A -5	19<br>118.2<br>118.2<br>190000-00-15 KI<br>1/15/25<br>-5 -5<br>-5 #N/A -5	4
5	25<br>119.2<br>119.2<br>190000-00-21 KI<br>1/15/25<br>-5 -5<br>-5 #N/A -5	26<br>119.3<br>119.3<br>190000-00-22 KI<br>1/15/25<br>-5 -5<br>-5 #N/A -5	27<br>119.4<br>119.4<br>190000-00-23 KI<br>1/15/25<br>-5 -5<br>-5 #N/A -5	28<br>119.5<br>119.5<br>190000-00-24 KI<br>1/15/25<br>-5 -5<br>-5 #N/A -5	29<br>119.6<br>119.6<br>190000-00-25 KI<br>1/15/25<br>-5 -5<br>-5 #N/A -5	30<br>#N/A 5	5

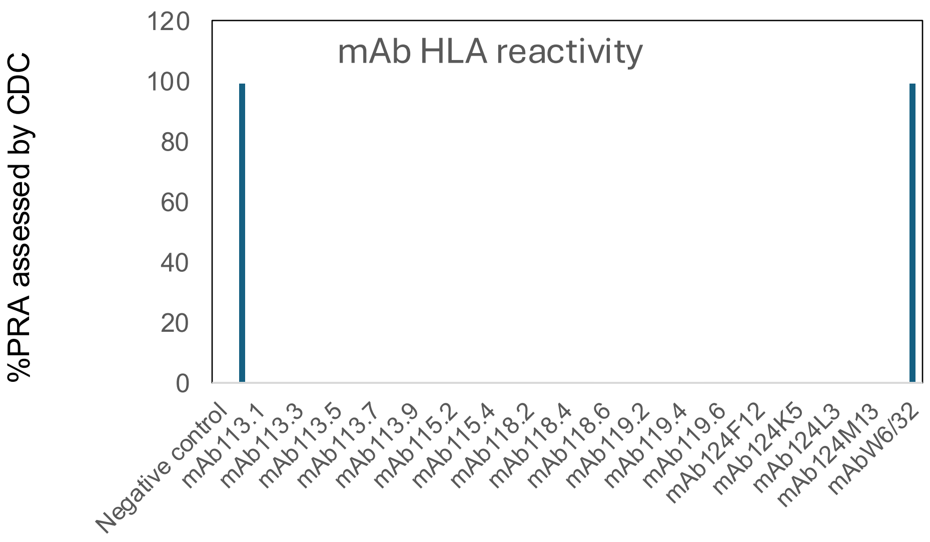

Figure S5

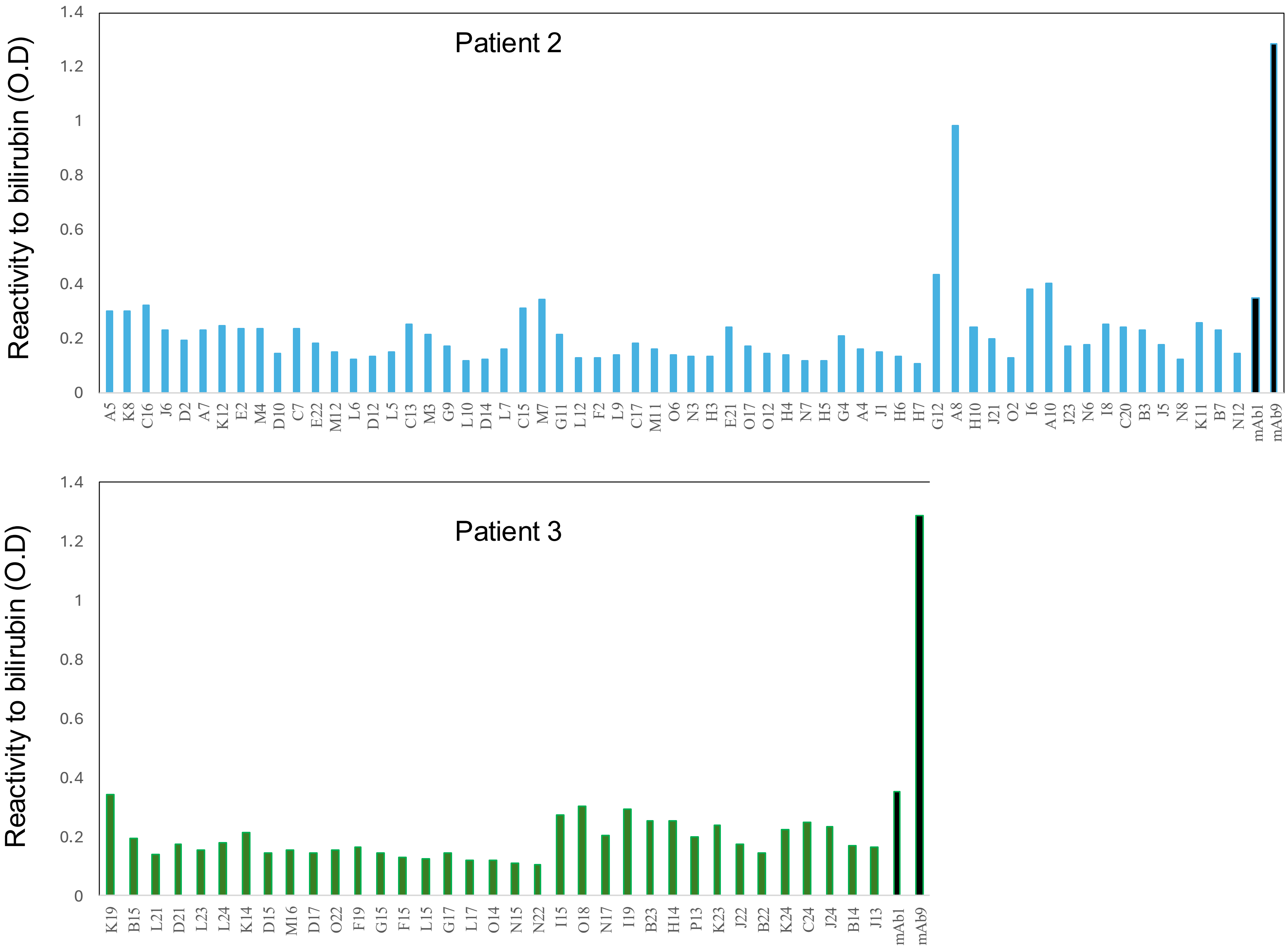
Patient 2
Reactivity to bilirubin (O.D)
0
0.2
0.4
0.6
0.8
1
1.2
1.4
A5 K8 C16 J6 D2 A7 K12 E2 M4 D10 C7 E22 M12 L6 D12 L5 C13 M3 G9 L10 D14 L7 C15 M7 G11 L12 F2 L9 C17 M11 O6 N3 H3 E21 O17 O12 H4 N7 H5 G4 A4 J1 H6 H7 G12 A8 H10 J21 O2 I6 A10 J23 N6 I8 C20 B3 J5 N8 K11 B7 N12 mAb1 mAb9
Patient 3
Reactivity to bilirubin (O.D)
0
0.2
0.4
0.6
0.8
1
1.2
1.4
K19 B15 L21 D21 L23 L24 K14 D15 M16 D17 O22 F19 G15 F15 L15 G17 L17 O14 N15 N22 I15 O18 N17 I19 B23 H14 P13 K23 J22 B22 K24 C24 J24 B14 J13 mAb1 mAb9

Figure S6

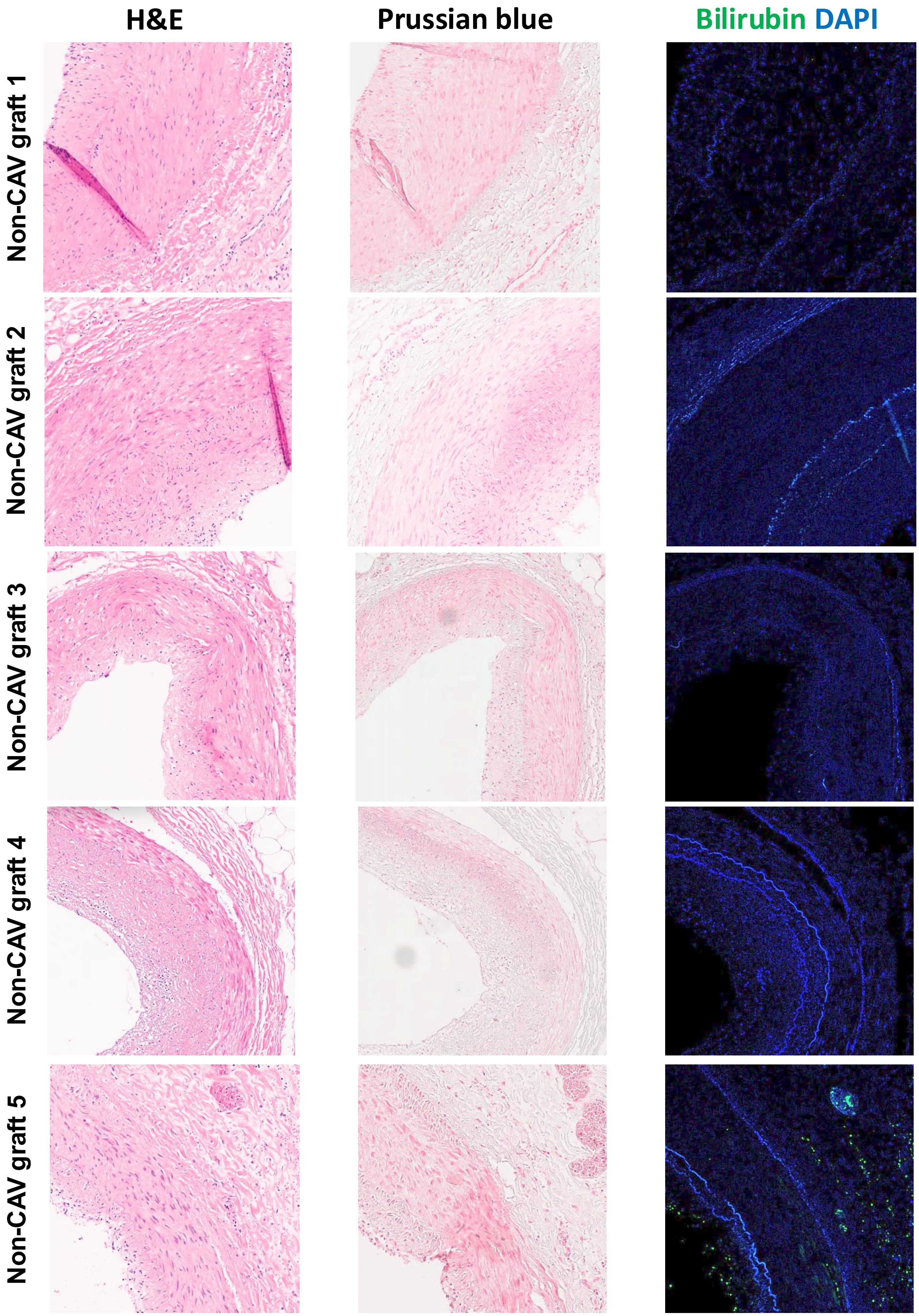

H&E
Prussian blue
Bilirubin DAPI
Non-CAV graft 1
Non-CAV graft 2
Non-CAV graft 3
Non-CAV graft 4
Non-CAV graft 5

Figure S7

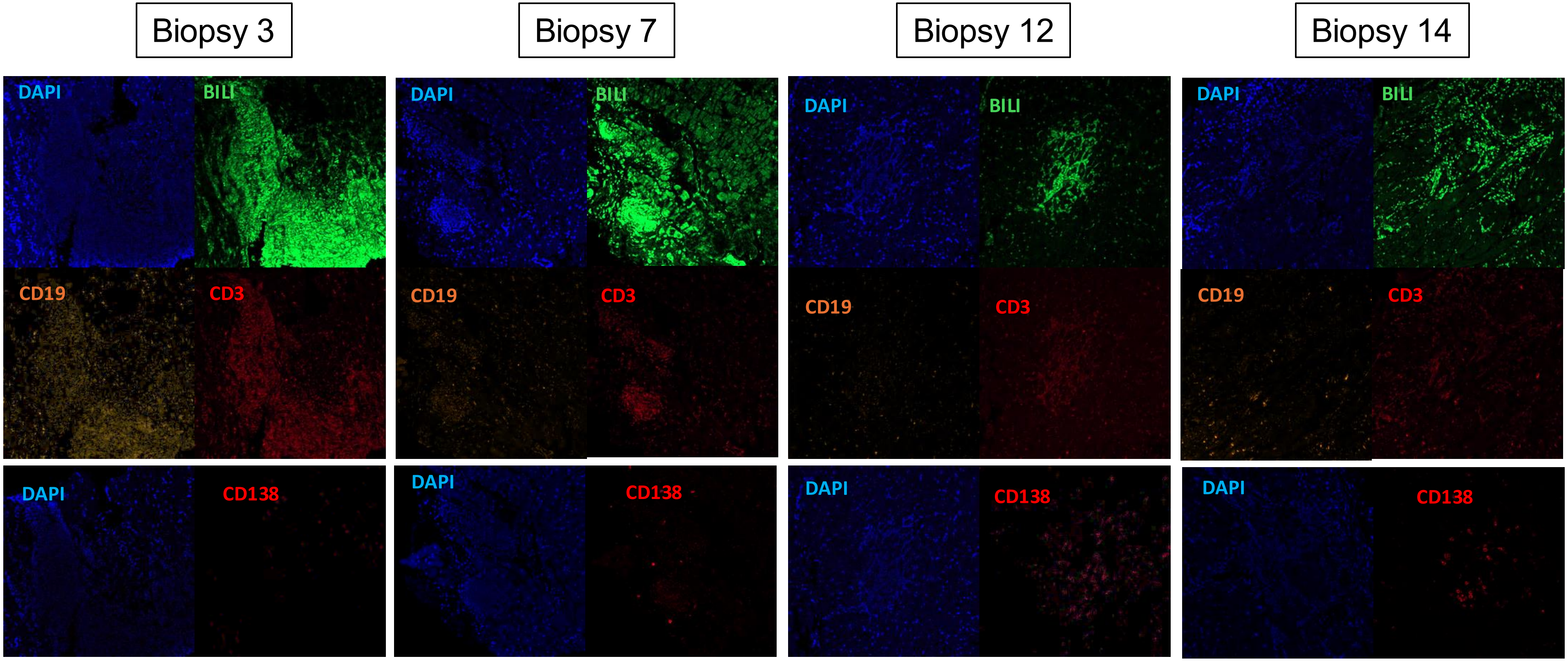
Biopsy 3
Biopsy 7
Biopsy 12
Biopsy 14
DAPI
BILI
CD19
CD3
DAPI
CD138

Figure S8

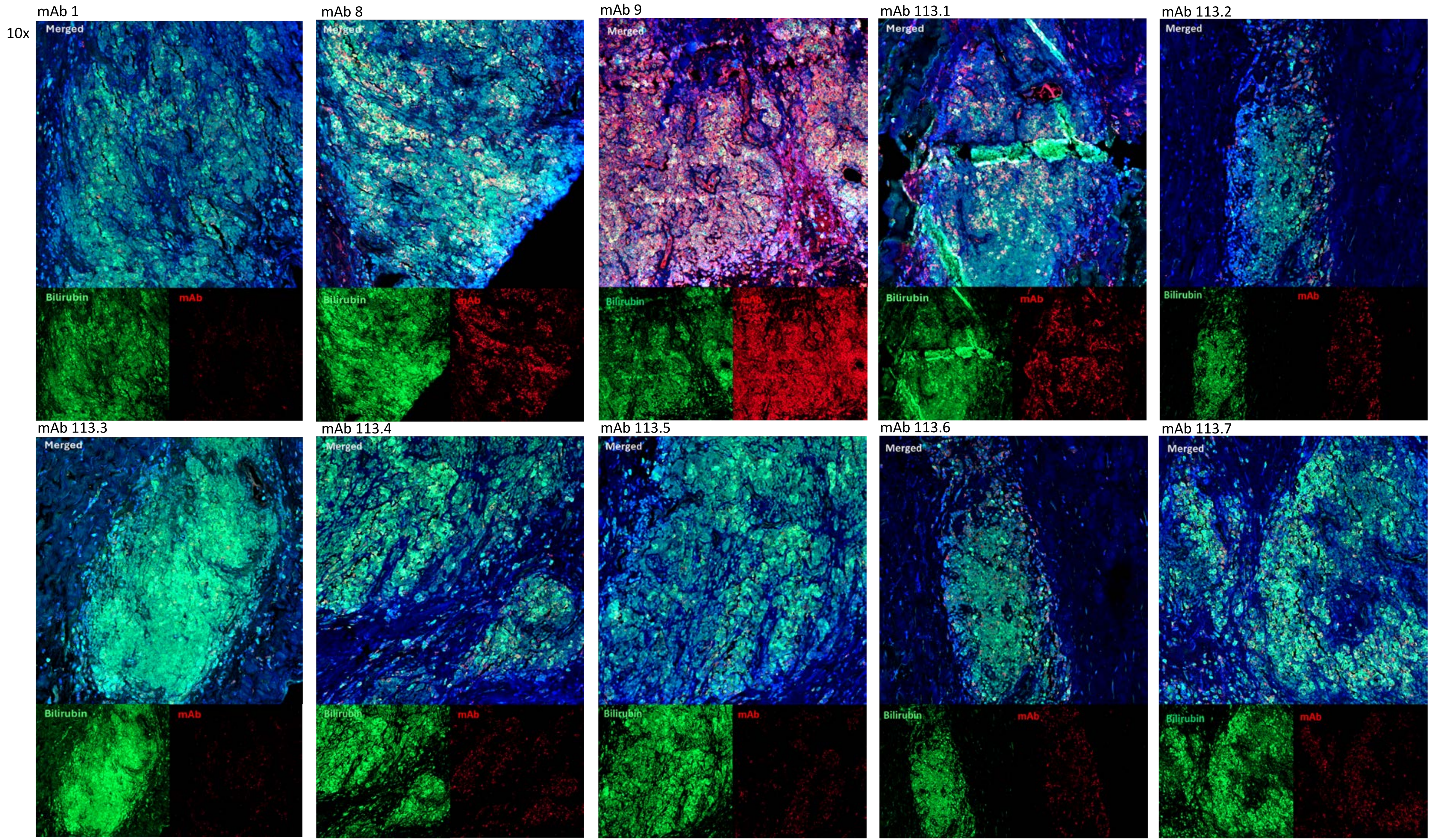
mAb 1
10x
Merged
Bilirubin
mAb
mAb 8
Merged
Bilirubin
mAb
mAb 9
Merged
Bilirubin
mAb
mAb 113.1
Merged
Bilirubin
mAb
mAb 113.2
Merged
Bilirubin
mAb
mAb 113.3
Merged
Bilirubin
mAb
mAb 113.4
Merged
Bilirubin
mAb
mAb 113.5
Merged
Bilirubin
mAb
mAb 113.6
Merged
Bilirubin
mAb
mAb 113.7
Merged
Bilirubin
mAb

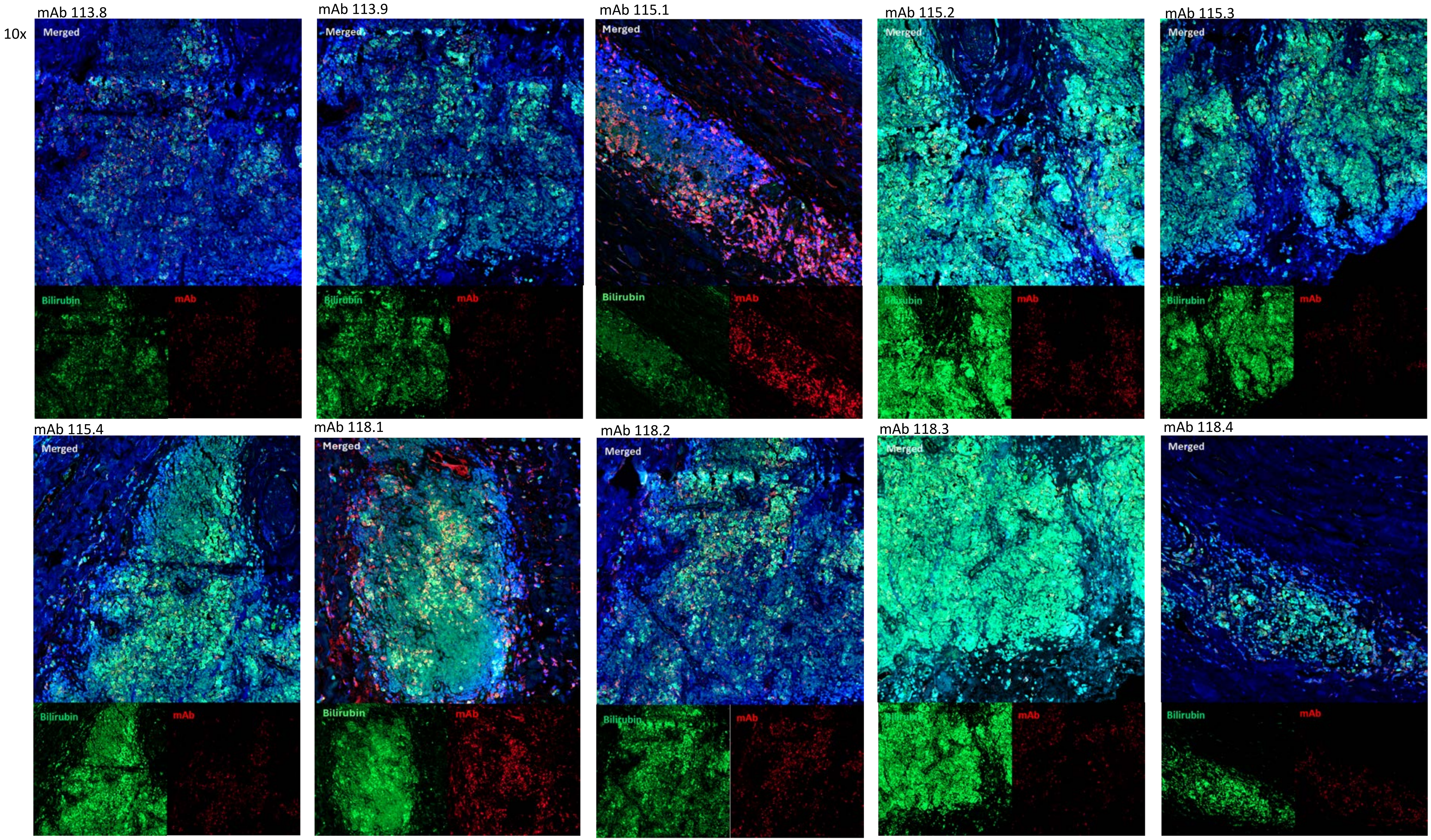
mAb 113.8
10x
Merged
Bilirubin
mAb
mAb 113.9
mAb 115.1
mAb 115.2
mAb 115.3
mAb 115.4
mAb 118.1
mAb 118.2
mAb 118.3
mAb 118.4

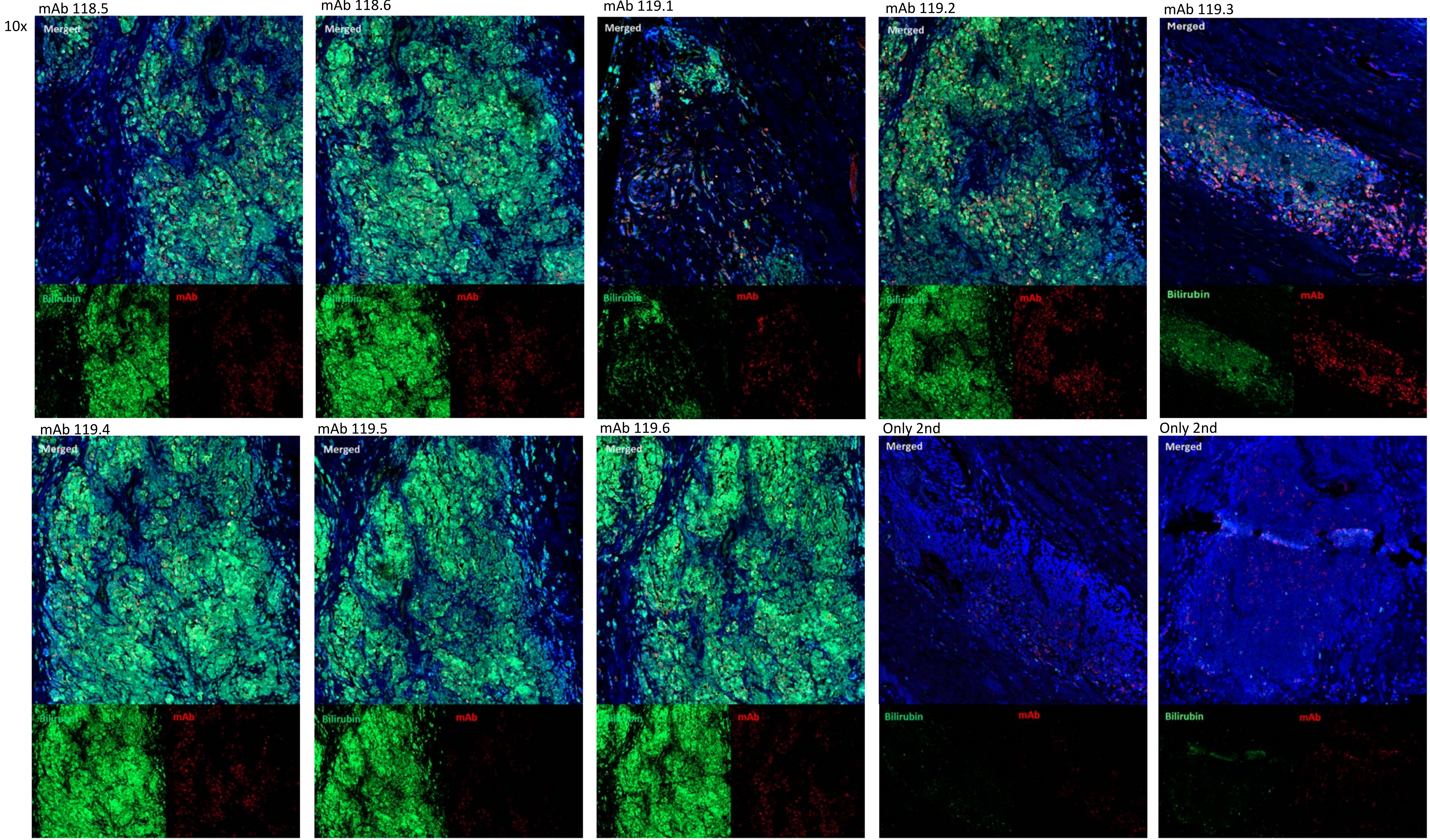
10x
mAb 118.5
Merged
Bilirubin
mAb
mAb 118.6
Merged
Bilirubin
mAb
mAb 119.1
Merged
Bilirubin
mAb
mAb 119.2
Merged
Bilirubin
mAb
mAb 119.3
Merged
Bilirubin
mAb
mAb 119.4
Merged
Bilirubin
mAb
mAb 119.5
Merged
Bilirubin
mAb
mAb 119.6
Merged
Bilirubin
mAb
Only 2nd
Merged
Bilirubin
mAb
Only 2nd
Merged
Bilirubin
mAb

Figure S9

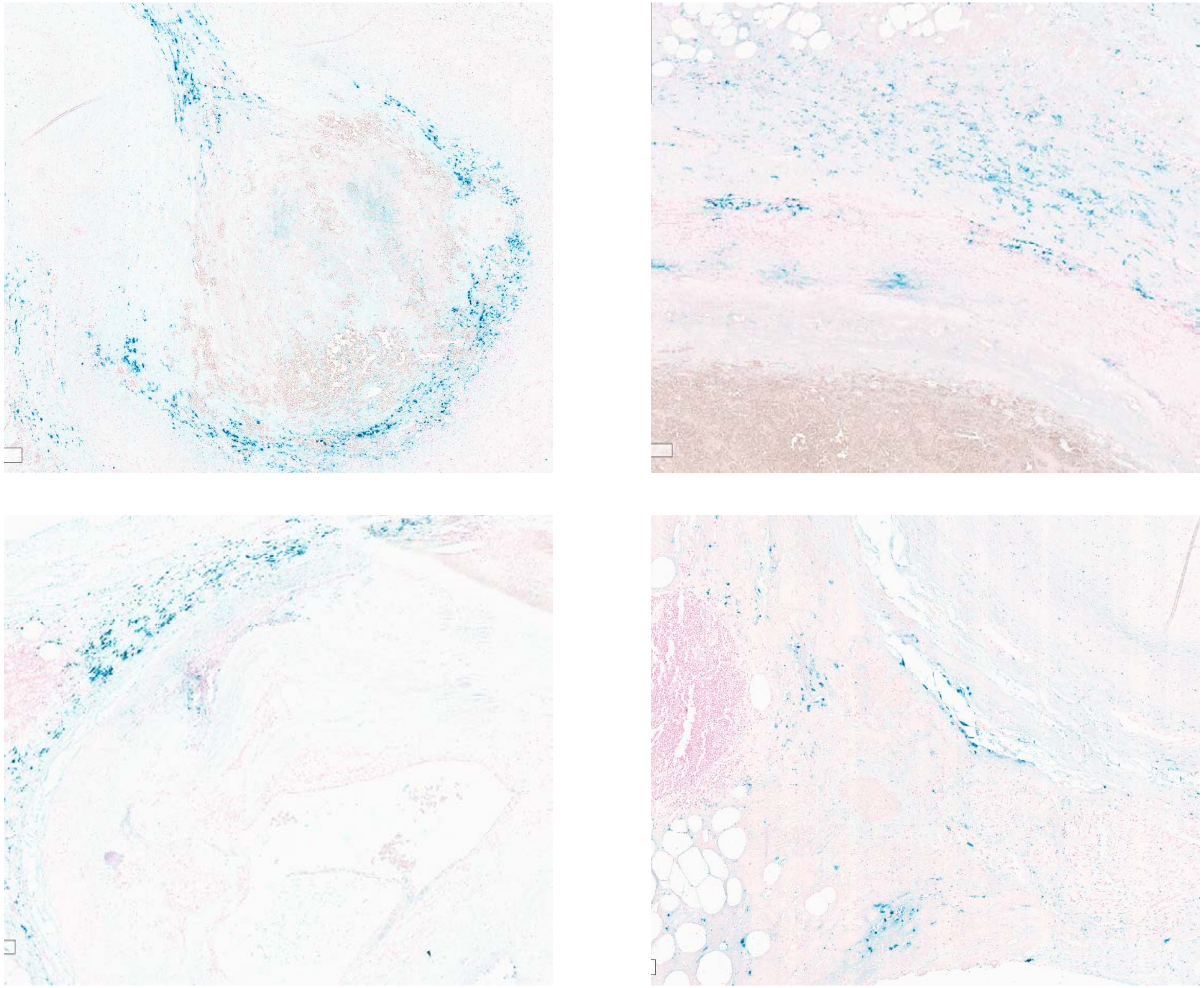

Figure S10

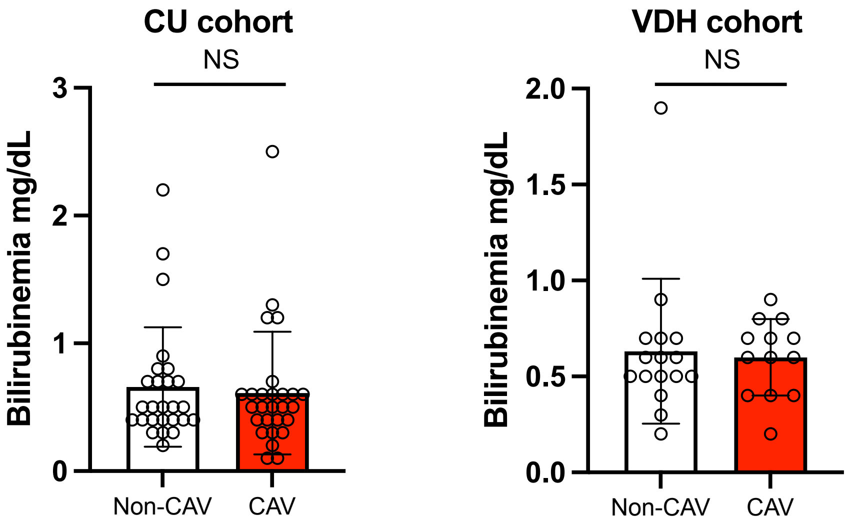
CU cohort
NS
3
2
1
0
Bilirubinemia mg/dL
Non-CAV
CAV
VDH cohort
NS
2.0
1.5
1.0
0.5
0.0
Bilirubinemia mg/dL
Non-CAV
CAV

Figure S11

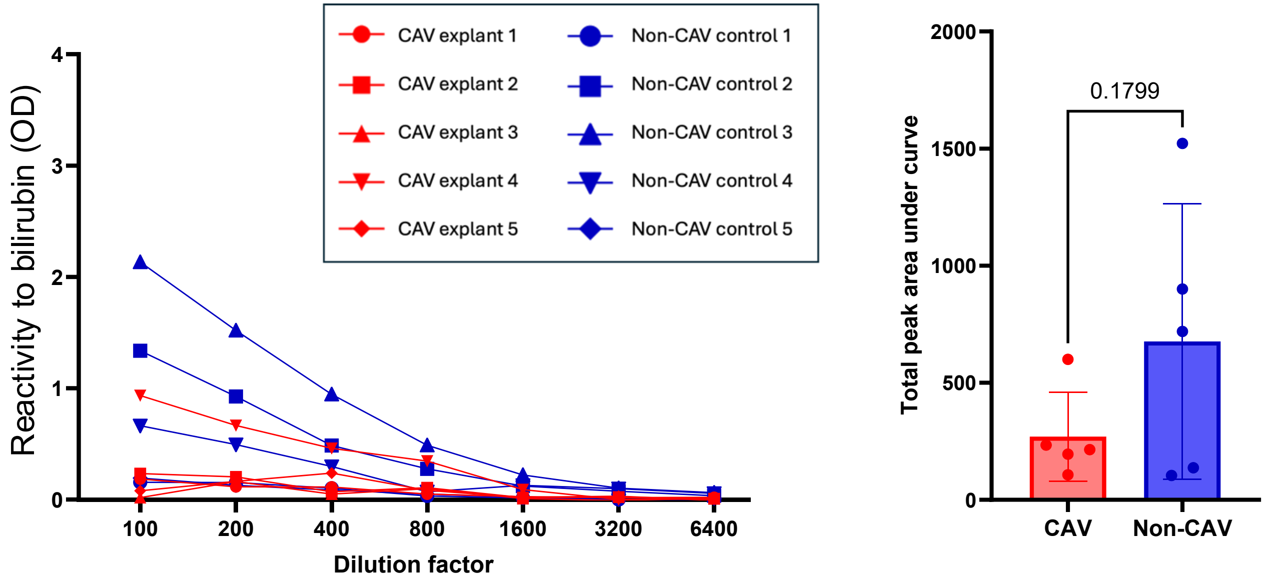

CAV explant 1
CAV explant 2
CAV explant 3
CAV explant 4
CAV explant 5
Non-CAV control 1
Non-CAV control 2
Non-CAV control 3
Non-CAV control 4
Non-CAV control 5
Reactivity to bilirubin (OD)
4
3
2
1
0
100
200
400
800
1600
3200
6400
Dilution factor
Total peak area under curve
2000
1500
1000
500
0
0.1799
CAV
Non-CAV

Figure S12
